# Ultrafast optical properties and applications of anisotropic 2D materials

**DOI:** 10.1515/nanoph-2023-0639

**Published:** 2024-01-17

**Authors:** Sang Ho Suk, Sung Bok Seo, Yeon Sik Cho, Jun Wang, Sangwan Sim

**Affiliations:** School of Electrical Engineering, Hanyang University, Ansan 15588, South Korea; Photonic Integrated Circuits Center, Key Laboratory of Materials for High-Power Laser, Shanghai Institute of Optics and Fine Mechanics, Chinese Academy of Sciences, Shanghai 201800, China

**Keywords:** anisotropic two-dimensional materials, ultrafast spectroscopy, carrier dynamics, exciton dynamics, ultrafast active all-optical modulation, pulse laser generation

## Abstract

Two-dimensional (2D) layered materials exhibit strong light-matter interactions, remarkable excitonic effects, and ultrafast optical response, making them promising for high-speed on-chip nanophotonics. Recently, significant attention has been directed towards anisotropic 2D materials (A2DMs) with low in-plane crystal symmetry. These materials present unique optical properties dependent on polarization and direction, offering additional degrees of freedom absent in conventional isotropic 2D materials. In this review, we discuss recent progress in understanding the fundamental aspects and ultrafast nanophotonic applications of A2DMs. We cover structural characteristics and anisotropic linear/nonlinear optical properties of A2DMs, including well-studied black phosphorus and rhenium dichalcogenides, as well as emerging quasi-one-dimensional materials. Then, we discuss fundamental ultrafast anisotropic phenomena occurring in A2DMs, such as polarization-dependent ultrafast dynamics of charge carriers and excitons, their direction-dependent spatiotemporal diffusion, photo-induced symmetry switching, and anisotropic coherent acoustic phonons. Furthermore, we review state-of-the-art ultrafast nanophotonic applications based on A2DMs, including polarization-driven active all-optical modulations and ultrafast pulse generations. This review concludes by offering perspectives on the challenges and future prospects of A2DMs in ultrafast nanophotonics.

## 1 Introduction

2D materials hold tremendous promise for advancing nano-device technologies owing to their excellent properties, such as atomic-scale dimensions, versatile functional heterostructures that allow for numerous combinations, remarkable flexibility, and a wide range of electronic structures (from insulators to semiconductors and metals) [1], [2]. In particular, researchers are actively exploring the potential of 2D materials in the fields of nanophotonics and optoelectronics, benefiting from their strong interactions with light and high compatibility with various device structures [3], [4], [5]. Their diverse optical spectra, which depend on the material type and thickness, further enhance their appeal in these domains. Additionally, many 2D materials are known for their ultrafast photo-induced dynamics, occurring on femtosecond and picosecond timescales. Such rapid responses not only facilitate the development of high-speed compact photonic devices but also offer broad operational bandwidths spanning from GHz to THz frequencies [6], [7], [8], [9].

In the realm of 2D materials, initial interest among researchers centered on substances with strong in-plane symmetry, such as graphene and MoS_2_. In 2014, black phosphorus (BP) emerged as a new 2D material of note due to its exceptional electrical properties and the high ability to tune bandgaps depending on thickness [10], [11], [12]. BP features a puckered hexagonal atomic arrangement, which results in lower in-plane symmetry and imparts notable anisotropy across its electrical, mechanical, and optical traits. Subsequently, a variety of A2DMs with different structures have been reported [8], [13], [14], [15], [16], [17], [18], [19], [20]. A2DMs typically refer to layered materials characterized by pronounced directional dependencies in physical properties due to their low crystallographic symmetry, encompassing various material groups such as rhenium dichalcogenides (ReS_2_ and ReSe_2_), quasi-one-dimensional layered materials (e.g., TiS_3_, ZrTe_5_), as well as BP. In particular, their diminished symmetry leads to strong dependences on light polarization, a fundamental property specifying the oscillation orientation of light waves. The polarization-dependent light-matter interactions hold both fundamental interest and practical value in areas including optical communications [21], bioimaging [22], neuromorphic systems [23], and information encryption [24]. A2DMs exhibit potential as active materials across these polarization-driven applications [8], [13], [14], [15], [16], [17], [18], [19], [20]. Notably, their ultrafast photoresponsivity renders A2DMs appealing candidates for developing polarization-controlled nanophotonic devices featuring functional degrees of freedom.

For these reasons, recent years have seen intensive investigations into the anisotropic optical properties of A2DMs and various related applications. Notably, there have been excellent review articles published including this subject [8], [13], [14], [15], [16], [17], [18], [19], [20]. However, there is a lack of review papers that specifically focus on the ultrafast optical properties and applications of A2DMs. In 2020, Liu et al. published an excellent review paper that centered on the nonlinear and ultrafast optical characteristics of A2DMs, along with their applications [8]. Since then, the interest in A2DMs has expanded significantly, revealing novel ultrafast anisotropic optical properties inherent to each material. Therefore, a current review and analysis of the latest research on the ultrafast optical properties and potential applications of A2DMs are warranted.

**Figure 1:**
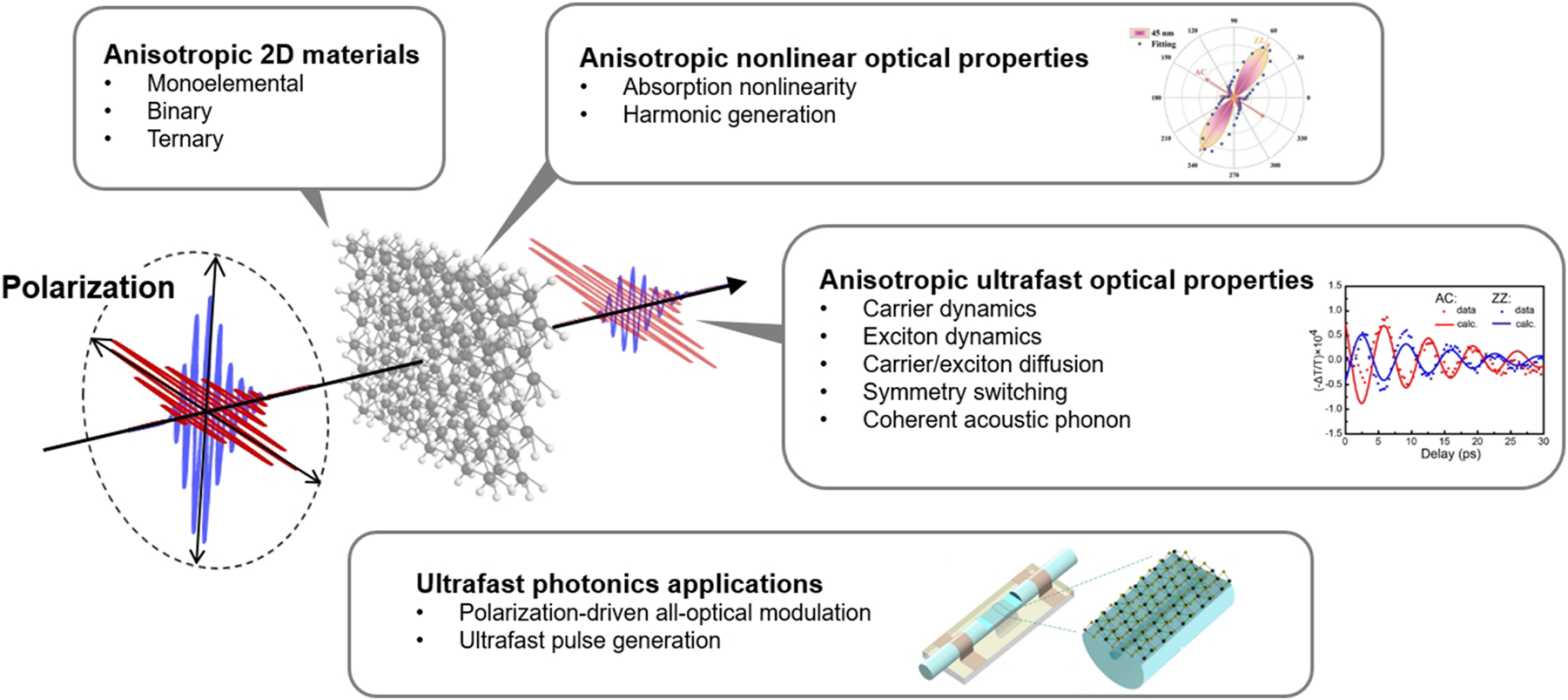
Ultrafast optical properties and applications of A2DMs. Reprinted figure with permission from [146] © 2021 Wiley-VCH GmbH. Reprinted with permission from [275]. Copyright 2021 American Chemical Society. Reprinted figure with permission from [294] © (CC-BY 4.0).

In this review, we discuss the recent anisotropic ultrafast optical phenomena and applications of A2DMs (Figure 1). Section 1 provides a background for this review. Section 2 categorizes various types of A2DMs and provides a brief overview of their atomic structural characteristics and the resulting anisotropic polarization-dependent linear optical absorption features. Subsequently, Section 3 discusses the anisotropic characteristics of nonlinear optical phenomena exhibited by A2DMs, such as absorption nonlinearity and harmonic generation. Then, Section 4 explores the diverse polarization-dependent ultrafast optical properties observed in A2DMs, studied by time-resolved spectroscopy. Specifically, we discuss the principles and types of transient absorption-based spectroscopic studies, upon which we review ultrafast anisotropic phenomena including polarization-dependent charge carrier and exciton dynamics, orientation-dependent diffusion, photo-induced symmetry switching, and polarization-dependent coherent acoustic phonons. We emphasize that these phenomena offer new insights in the context of ultrafast anisotropic light-matter interactions, yet they have rarely been covered in other reviews relevant to A2DMs. Section 5 elaborates on representative ultrafast photonics applications rooted in A2DMs, such as ultrafast active all-optical modulation driven by polarization and ultrafast pulse laser generation. Finally, Section 6 presents challenges and prospects in anisotropic ultrafast optical research based on A2DMs, concluding this review.

## 2 Anisotropic 2D materials and their linear absorption properties

In this section, we discuss the distinct anisotropic atomic structures of various A2DMs and their fundamental polarization-dependent absorption properties. Please note that Raman spectroscopy also effectively reveals the anisotropic optical properties of A2DMs [8], [13], [15], [20]. However, we primarily focus on the absorption properties, which is more directly connected to the ultrafast optical phenomena to be mainly reviewed later in this article. In the commonly utilized range from infrared (IR) to ultraviolet (UV) for material characterization through optical absorption, interband transitions typically play a pivotal role. Following Fermi’s Golden Rule, the absorption coefficient (*α*) is proportional to [25]

(1) $$\alpha \propto \underset{i,f}{\sum }{\left|M_{if}\right|}^{2}\delta \left(E_{f}-E_{i}-E\right),$$

where subscripts *i* and *f* denote the initial and final states involved in each transition, and *M*_*if*_ represents the transition matrix element. Under the dipole approximation, the relationship *M*_*if*_ ∝ ***D*** ⋅ ***P***_*L*_ holds, where $D=\left\langle f|\nabla |i\right\rangle$ represents the dipole vector determined by the material’s electronic structure, and ***P***_*L*_ signifies the polarization vector of light. In A2DMs, the inner product of ***D*** and ***P***_*L*_ can vary with the angle between a specific crystal axis and light polarization, resulting in anisotropic absorption coefficients. Polarization-dependent photoluminescence (PL) is also widely employed for the characterization of anisotropic linear optical properties of A2DMs. PL is determined by the same polarization-dependent matrix elements as interband absorption [20]. However, unlike interband absorption, which can sensitively detect transitions between higher-lying states, PL predominantly observes radiative recombination of electrons and holes at the edges of conduction band (CB) and valence band (VB) or lower-lying defect states.

**Figure 2:**
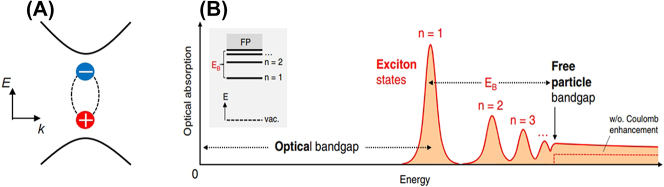
Excitons in 2D semiconductors. (A) Illustration of an exciton in a semiconductor. (B) An illustration depicting an optical absorption spectrum of a 2D semiconductor, featuring a sequence of prominent exciton resonances situated below the free particle bandgap. Reprinted figure with permission from [27]. Copyright 2018 by the American Physical Society.

In the absorption and emission of semiconducting 2D materials, excitons play a vital role as electron-hole pairs bound by Coulomb interaction (Figure 2A) [26], [27], [28]. Excitons exhibit a Rydberg series akin to hydrogen atoms, resulting in peak formations in the absorption spectrum, as illustrated in Figure 2B [27]. Here, the free-particle bandgap (or quasiparticle bandgap) appears as the onset of unbound electron–hole absorption, manifesting as the onset of the continuum in the spectrum. The lowest exciton state labeled with *n* = 1, appearing below the free-particle bandgap, determines the optical bandgap. The difference between the free-particle bandgap and the optical bandgap corresponds to the exciton binding energy (indicated as *E*_*B*_ in Figure 2B) [27], a crucial parameter that determines the exciton’s resonance energy, stability, and interactions with various exciton species. In 2D materials, the strength of Coulomb interaction is significantly enhanced compared to their bulk counterparts due to weak dielectric screening and strong spatial confinement effects. Consequently, excitons in 2D semiconductors possess high binding energies of several hundred meV, often resulting in pronounced excitonic effects even at room temperature. Excitons appearing in A2DMs exhibit strong exciton binding combined with high polarization-dependent anisotropy. In this section, we highlight the polarization-dependent anisotropy of observed excitons in some semiconducting A2DMs. Other characteristics of excitons in A2DMs, such as binding energies and their thickness dependences, are well summarized in recent publications [20].

Below we categorize A2DMs based on their chemical formulas [18] and discuss the atomic structures as well as experimentally revealed anisotropic linear absorption, emission, and excitonic properties for each. Optical anisotropy in 2D materials can also be achieved through van der Waals hetero-stacking between isotropic/anisotropic [14], [29] or anisotropic/anisotropic [30] 2D materials, as well as through strain engineering [31] and nanopatterning [32] of isotropic materials. However, this review predominantly focuses on the characteristics of individual A2DMs that have been investigated mainly for their ultrafast polarization-dependent optical phenomena so far.

### 2.1 Monoelemental materials

**Figure 3:**
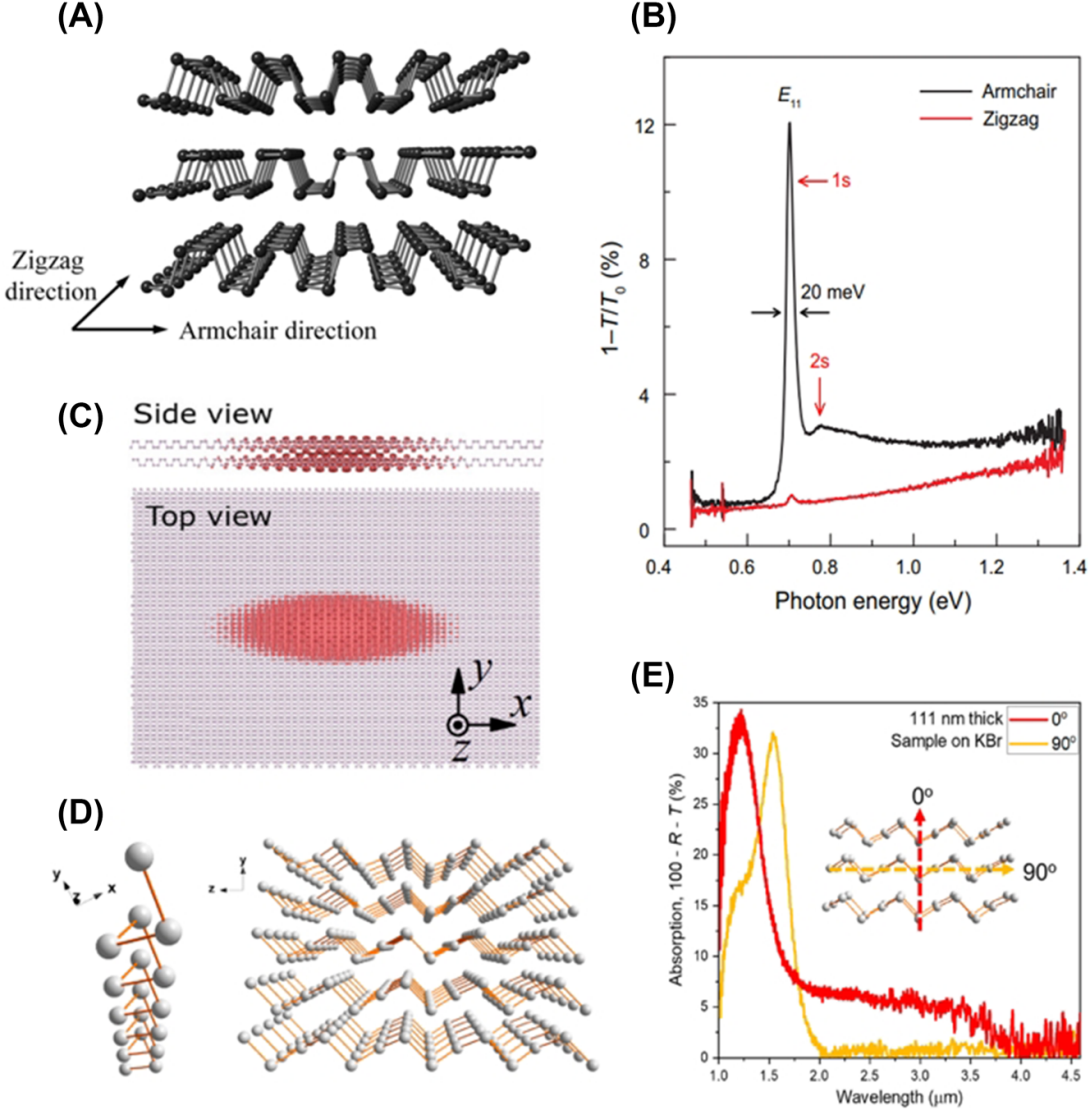
Monoelemental A2DMs. (A) Atomic structure of BP Reprinted figure with permission from [33] © The Royal Society of Chemistry 2015. (B) Polarization-resolved extinction spectra of four-layer BP [44] © (CC BY-NC). (C) Exciton wavefunction in bilayer BP. Reprinted figure with permission from [45]. Copyright 2020 by the American Physical Society. (D–E) Atomic structure (D) and polarization-dependent absorption spectra (E) for Te (Reprinted with permission from [50]. Copyright 2018 American Chemical Society).

BP with a distorted orthorhombic structure is the most extensively studied A2DM [33], [34], [35], [36]. Within each plane of BP, phosphorus atoms form a twisted hexagonal structure through covalent bonds, resulting in a puckered shape. Consequently, as depicted in Figure 3A, the armchair (AC) morphology along a direction is distinctly different from the perpendicular zigzag (ZZ) shape, leading to anisotropic properties [33]. BP’s interband absorption demonstrates linear dichroism, showing higher overall absorption for AC polarization compared to that along ZZ polarization. Additionally, the optical absorption edges in AC polarization are observed at energies less than half of those in ZZ polarization [11]. BP possesses direct-gap nature, thus exhibits efficient PL [37]. Similar to absorption anisotropy, BP emits light with AC-polarized direction. Emission from defect states in BP is also AC-polarized [38].

Few-layer and thinner BPs exhibit remarkable exciton features [25], [39], [40], [41], [42], [43], [44], [45], [46]. The excitons in BP are strongly polarized in the AC direction, similar to the anisotropy of interband absorption. Figure 3B depicts polarization-dependent absorption spectra observed in a 4-layer BP on a PDMS substrate, revealing 1 s and 2 s exciton states when light polarization is AC [44]. In contrast, a relatively small response is observed for ZZ polarization. These anisotropic characteristics align with the highly elongated shape of the exciton wavefunction along the AC direction (Figure 3C) [45]. The energy of BP excitons exhibits significant thickness dependence due to interlayer interactions. While the monolayer BP’s optical bandgap is approximately 1.69 eV, the optical bandgap decreases monotonically as thickness increases, reaching around 0.34 eV for bulk thickness, covering a wide spectral range through thickness engineering [36]. In addition, the exciton binding energy is over 300 meV in monolayer BP, but it gradually decreases with increasing thickness, falling below 100 meV in structures thicker than 6-layers [44]. Further details on the BP excitons and associated discovery history are extensively summarized in recent reviews [20], [36]. A similar atomistic structure to BP is found in monoelemental black arsenic (b-As), which has recently gained attention due to its strong electronic and optical anisotropy [47], [48].

In addition to BP and b-As with their distorted hexagonal structures, various anisotropic monoelemental A2DMs such as tellurium (Te) [49], [50], [51] and fibrous red phosphorus [52] are being investigated. As an example, Te crystals exhibit an intriguing atomic arrangement where adjacent Te atoms form covalent bonds to create one-dimensional (1D) helical chains (Figure 3D) [50]. Thus, Te is a 1D system, but quasi-2D nanosheets of Te have also been achieved through various methods such as hydrothermal synthesis and liquid phase exfoliation [49]. Figure 3E displays the polarization-dependent absorption spectrum measured from a solution-synthesized quasi 2D Te nanoflake with a thickness of 111 nm, revealing significant anisotropy in the short-wave infrared region of 1.4–3 μm [50].

### 2.2 Binary materials

There exist various types of binary A2DMs composed of two different elements. Thus far, extensive research has been conducted on materials with stoichiometries in the forms of AB, AB_2_, and AB_3_. In this section, we focus on these materials and briefly discuss other types of binary A2DMs as well.

#### 2.2.1 AB-type

**Figure 4:**
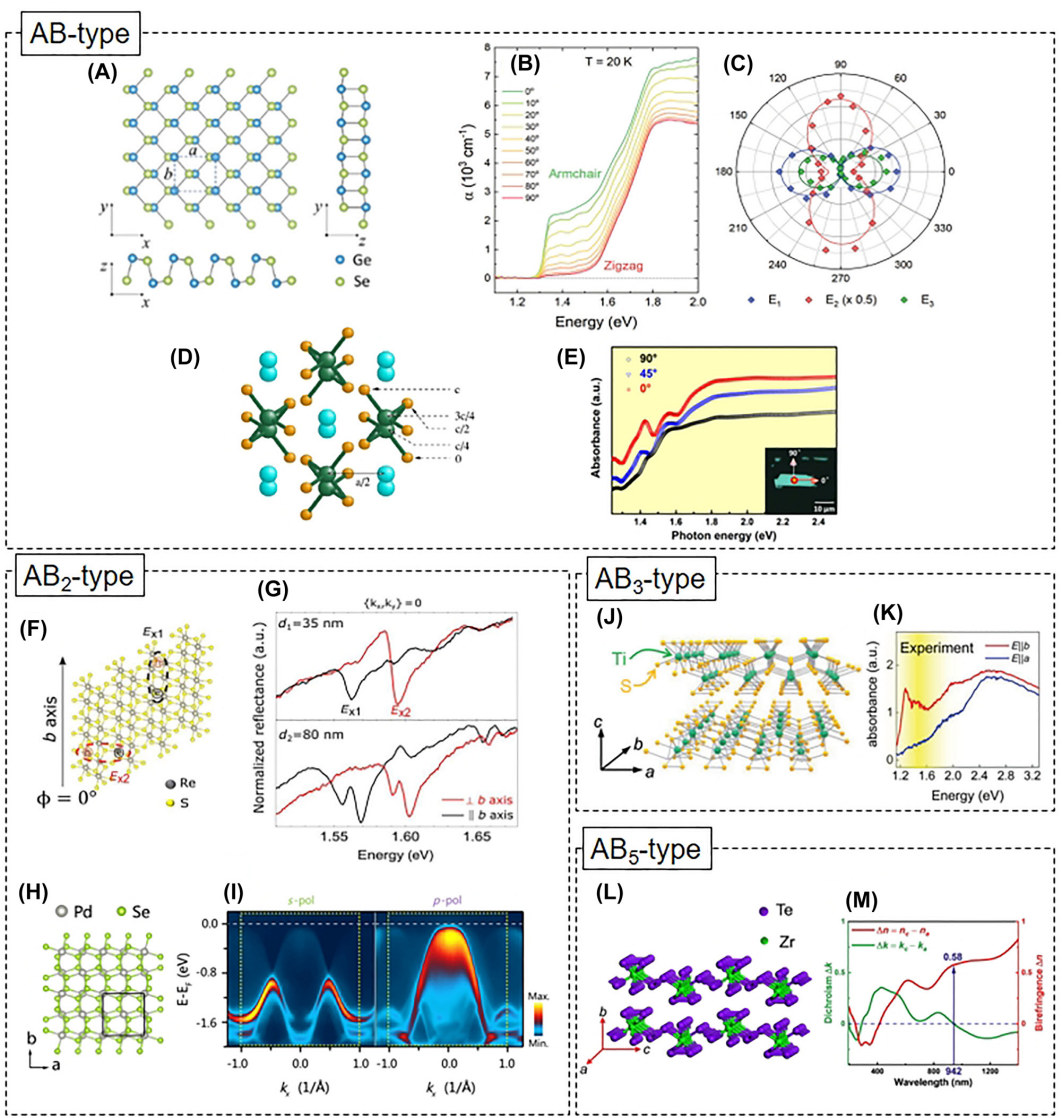
Binary A2DMs. (A–B) Atomic structure (A) and polarization-dependent absorption coefficient (B) of GeSe. Reprinted figure with permission from [62] © The Royal Society of Chemistry 2021. (C) Polarization-dependent oscillator strength of three interband transitions in GeSe Reprinted figure with permission from [62] © The Royal Society of Chemistry 2021. (D) Atomic structure of TlSe. Reprinted figure with permission from [63]. Copyright 2004 by the American Physical Society. (E) Polarization-dependent absorption spectra for a TlSe flake. Reprinted with permission from [64]. Copyright 2018 American Chemical Society. (F–G) Top-view atomic structure of ReS_2_ (F) and polarization-resolved reflectance spectra for two different thicknesses (G) [81] © (CC BY). (H–I) Atomic structure (H) and polarization-resolved ARPES spectra (I) in PdSe_2_ . Reprinted figure with permission from [89]. Copyright 2022 by the American Physical Society. (J–K) Atomic structure of TiS_3_ (J) and its polarization-dependent absorbance (K). Reprinted figure with permission from [102] © 2023 Wiley-VCH GmbH. (L–M) Atomic structure (L) and wavelength-resolved dichroism and birefringence of ZrTe_5_ (M). Reprinted with permission from [107]. Copyright 2021 American Chemical Society.

Prominent examples of AB-type binary A2DMs include group-IV monochalcogenides such as SnS [53], [54], SnSe [55], [56], GeS [57], [58], [59], [60], and GeSe [61], [62]. These materials share a puckered orthorhombic structure similar to BP, resulting in high anisotropic optical absorption properties. However, unlike BP, this group of materials holds a crucial advantage in terms of high air stability, which is a significant advantage for real applications. As an example of anisotropic group-IV monochalcogenides, Figure 4A illustrates the puckered atomic structure of GeSe, revealing in-plane AC direction (*x*-axis) and ZZ direction (*y*-axis) [62]. Tołłoczko et al. observed similar polarization-dependent absorption behavior to BP, where GeSe exhibited higher overall absorption in AC polarization compared to ZZ polarization (Figure 4B) [62]. Furthermore, the authors resolved three anisotropic direct transitions with strong polarization dependencies, *E*_1_ at 1.29 eV, *E*_2_ at 1.52 eV, and *E*_3_ at 1.58 eV, above the indirect fundamental gap of 0.99 eV, using photoreflectance measurements and theoretical analysis. Interestingly, as depicted in Figure 4C, while *E*_1_ (blue dots) and *E*_3_ (green dots) transitions are allowed in AC polarization, the *E*_2_ transition (red dots) exhibits an opposite polarization dependence, showing a strong response when light is polarized in the ZZ direction [62].

In addition to group-IV monochalcogenides, attention has been also drawn to group-III monochalcogenides, such as TlSe [63], [64] and GaTe [65], [66]. Figure 4D illustrates the low-symmetry structure of TlSe, where negatively charged 1D chains oriented along the [001] direction composed of Tl^3+^ (green dots) and Se^2−^ (orange dots) are combined with positively charged Tl^+^ (cyan dots) [63]. Due to this quasi-1D nature, TlSe 2D flakes exhibit elongated shapes along the [001] direction and display higher absorption at the polarization along [001] (Figure 4E) [64]. Monoclinic GaTe also possesses an anisotropic structure with chain-like arrangements [65]. However, the optical absorption in GaTe is known to have relatively lower anisotropy [66]. GaTe’s electron structure and optical properties are significantly affected by oxidation in ambient conditions, prompting research into various passivation methods [67], [68], [69]. Furthermore, group-IV monopnictides such as SiP [70], GeP [71], SiAs [72], and GeAs [73] are also gaining attention due to their anisotropic atomic structures and unique optical polarization-dependent properties.

#### 2.2.2 AB_2_-type

Rhenium dichalcogenides (ReX_2_; where X = S and Se) are one of the representative AB_2_-type A2DMs classified as group-VII transition metal dichalcogenides (TMDs) [74], [75], [76], [77], [78], [79]. ReX_2_ has a distorted triclinic atomic structure in the 1T phase. These materials have gained renewed attention due to the discovery that they exhibit properties similar to monolayers even in bulk crystals, due to weak interlayer coupling [80]. Within each layer, rhenium atoms form ZZ-shaped 1D chains along the crystal *b*-axis, resulting in low in-plane symmetry (Figure 4F) [81]. In ReX_2_, anisotropic in-plane exciton states with different orientations have been observed [77], [82], [83], [84]. The upper panel of Figure 4G shows the lowest two excitons in ReS_2_, labeled as *E*_*x*1_ and *E*_*x*2_, as spectral dips measured by polarization-dependent reflectance [81]. Here, *E*_*x*1_ is strong when polarized parallel to the *b*-axis, while *E*_*x*2_ is pronounced when polarized perpendicular to the *b*-axis. The distinct polarization directions of these two excitons are also illustrated in Figure 4F. In addition to these two resonances, as shown in the bottom panel of Figure 4G, additional anisotropic resonances or resonance splitting has been observed [81], which has been a significant topic of debate. This has been attributed to various factors such as close-lying bright exciton states [85], neutral-donor-bound-exciton by trapping polarized free excitons [84], singlet-triplet splitting [86], internal reflections within the flake [87], and the longitudinal exciton feature [81]. Apart from these issues, there have also been various debates related to whether the interlayer coupling is weak or strong and the direct/indirect nature of the fundamental gap in ReX_2_. For details on these matters, reference can be made to other review articles [20], [77].

Palladium dichalcogenides (PdX_2_; where X = S and Se) are also representative anisotropic material with high air stability. PdSe_2_ shown in Figure 4H possesses a unique anisotropic puckered pentagonal structure, leading to polarization-dependent interactions with light [88]. Recently, angle-resolved photoemission spectroscopy and theoretical calculations have revealed that PdSe_2_ exhibits significant linear dichroism due to its orbital-dependent valence band configurations (Figure 4I) [89]. Germanium dichalcogenides (GeX_2_; where X = S and Se) and germanium dipnictides (e.g., GeAs_2_) are also drawing attention due to their intriguing anisotropic properties [90]. Compared to orthorhombic GeS and GeSe, monoclinic GeX_2_ has a relatively higher bandgap. Yan et al. demonstrated that monolayer GeSe_2_ with a wide bandgap of 2.96 eV exhibits anisotropic absorption, enabling polarization-selective UV detection [91]. Recently, Lee et al. observed strong and weak anisotropic PL peaks at 2.5 eV and 1.8 eV, respectively, from GeSe_2_ grown via metal-organic chemical vapor deposition (CVD) [92]. These peaks are attributed to band-edge transitions and emission by mid-gap states due to oxygen-passivated selenium vacancies, respectively.

#### 2.2.3 AB_3_-type

Transition metal trichalcogenides (TMTs), which include TiS_3_, TiSe_3_, ZrS_3_, ZrSe_3_, ZrTe_3_, HfS_3_, and HfSe_3_, are representative AB_3_-type anisotropic nanomaterials [93], [94], [95]. While these materials share similar monoclinic quasi-1D structures, they can be categorized into three cases: TiS_3_-type, ZrSe_3_-type, or both [96]. Among the TMT materials, let us examine TiS_3_, which has relatively more research focus so far [97], [98], [99], [100], [101]. As seen in Figure 4J [102], each TiS_3_ layer forms 1D chains along the crystal *b*-axis composed of trigonal prisms with Ti centers and S vertexes. Consequently, exfoliated TiS_3_ flakes typically have elongated needle-like shapes [103]. TiS_3_ exhibits linear dichroism with strong interband transitions around 1.2–1.3 eV when the polarization of light is parallel to the 1D chains along *b*-axis (Figure 4K) [97], [98], [99], [100], [101], [102]. The excitons in TiS_3_ were observed at 0.91 eV through PL experiments, and they also show polarization-dependent behavior, particularly along the *b*-axis [99]. Anisotropic excitons in other TMTs are also noteworthy. ZrS_3_ and ZrSe_3_ exhibit anisotropic excitons that manifest in polarization along the crystal 1D chain direction [104]. Recently, it has been demonstrated that exciton resonances in few-layer ZrSe_3_ are effectively modulated by strain induced along the 1D chain [105]. Additionally, anisotropic TMTs with different atomic structures such as MoO_3_ are being intensively investigated [106].

#### 2.2.4 Others

In addition to the discussed AB, AB_2_, and AB_3_ types, other A2DMs with different stoichiometries are also being studied. As an example, the AB_5_-type narrow-gap ZrTe_5_ exhibits a unique crystal structure containing quasi-1D Zr–Te chains formed along the *a*-axis (Figure 4L) [107], resulting in electrical [108], thermal [109], and optical anisotropy [107]. While this material has primarily garnered attention for its intriguing topological features [110], [111], [112], [113], recent findings from Mueller matrix spectroscopic ellipsometry have revealed high birefringence and linear dichroism spanning the UV-to-near-infrared (NIR) range (Figure 4M) [107]. Moreover, other types of binary materials such as Si_2_Te_3_ [114], As_2_S_3_ [115] and In_4_Se_3_ [116] are also drawing attention due to their unique anisotropic structures and properties.

### 2.3 Ternary materials

Recently reported ternary compounds such as In_2_SnS_4_ [117], Nb_3_SeI_7_ [118], GaPS_4_ [119], ZrGeTe_4_ [120], Ta_2_NiS_5_ [121], and TaIrTe_4_ [122] exhibit unique anisotropic structures along with high optical dichroism and optoelectronic functionalities. For example, ZrGeTe_4_ nanoribbon-based photodetectors have demonstrated high photoresponsivity and polarization selectivity across a broad range from visible to NIR [120]. In addition, manipulating the stoichiometric ratio has been used to control physical properties in anisotropic ternary materials, such as Ge_1−*x*_Sn_*x*_Se_2_ [123], SnS_1−*x*_Se_*x*_ [124], and ReS_2−*x*_Se_*x*_ [125]. Particularly in SnS_1−*x*_Se_*x*_, symmetry breaking due to composition control and corresponding changes in optical and thermoelectric properties have been observed [124]. TaIrTe_4_, revealed as a layered Weyl semimetal, exhibits distinctive topological features [126]. We will review recently reported anisotropic ultrafast dynamics of this material in mid-infrared (MIR) [122].

## 3 Anisotropic nonlinear optical properties

In the interaction between light and matter, the polarization *P* of a material is given as follows [127]:

(2) $$P=\varepsilon_{0}\left(\chi^{\left(1\right)}E+\chi^{\left(2\right)}E^{2}+\chi^{\left(3\right)}E^{3}+\cdots \right).$$

Here, *ɛ*_0_ represents the vacuum permittivity, $\chi^{\left(n\right)}$ corresponds to the *n*-th order susceptibility, and *E* denotes the intensity of the electric field of light. In the case of weak light intensity, only the first linear term on the right-hand side is considered, and the remaining terms can be approximately neglected. However, as the light intensity becomes sufficiently strong, higher-order terms become significant, giving rise to various nonlinear optical effects. The second-order term ( $\chi^{\left(2\right)}E^{2}$ ) leads to phenomena such as second harmonic generation (SHG), the Pockels effect, and optical rectification, while the third-order term ( $\chi^{\left(3\right)}E^{3}$ ) induces phenomena like third harmonic generation (THG), saturable absorption (SA), and the Kerr effect. Each of these phenomena holds fundamental significance and offers high applicability. Notably, second-order effects occur only when a material lacks inversion symmetry, whereas there are no such restrictions for third-order effects. Anisotropic second-order and third-order effects have been observed in A2DMs. These phenomena are crucial not only for applications such as polarization-driven optical modulation but also for identifying crystal directions. In this section, we focus on the anisotropic behaviors of absorption nonlinearity and harmonic generation, which are extensively studied effects in A2DMs. For a more comprehensive review of nonlinear optical effects in 2D materials, including isotropic materials, we refer readers to recently published excellent review articles [4], [20], [128].

### 3.1 Absorption nonlinearity

**Figure 5:**
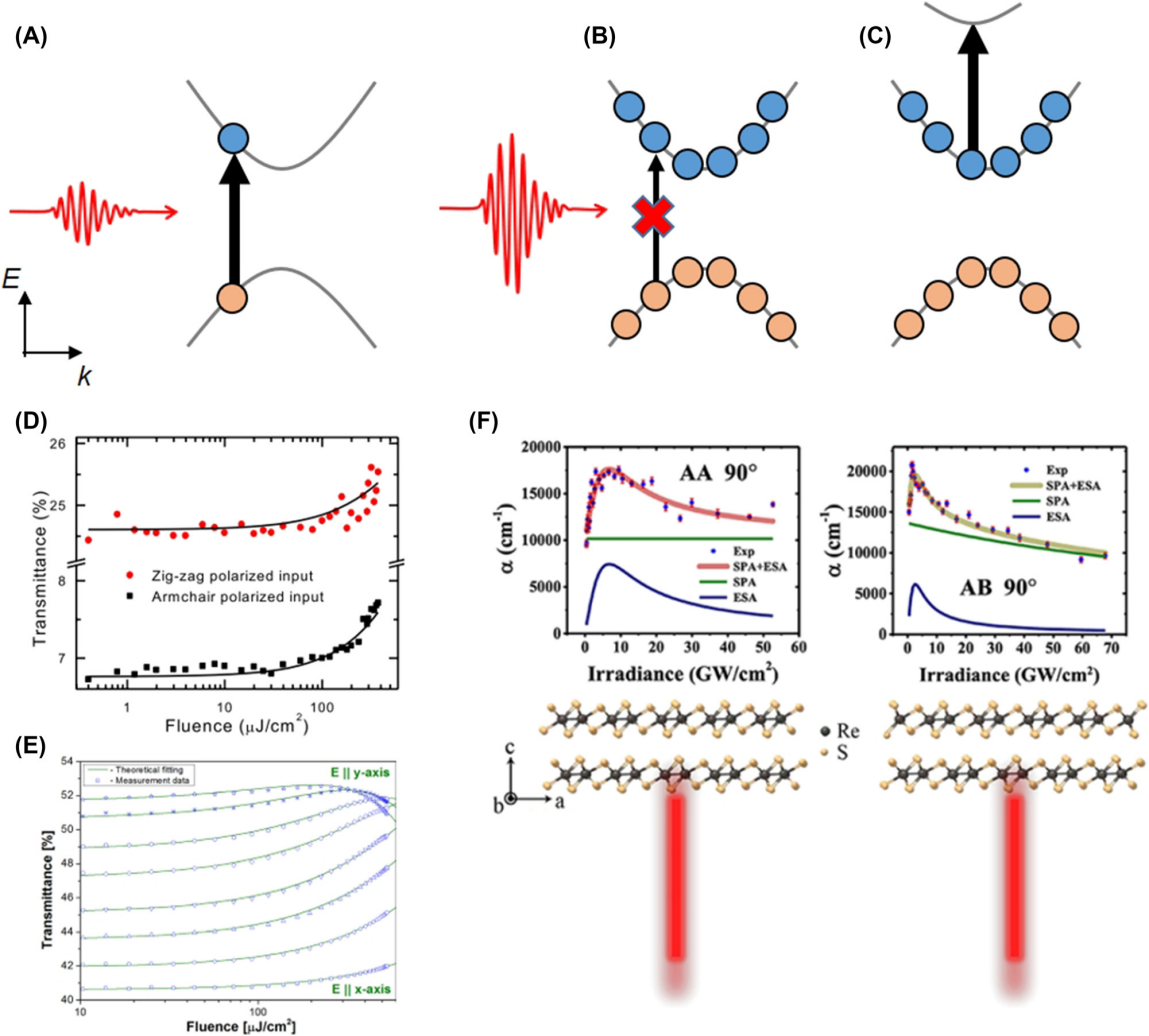
Nonlinear absorption properties in A2DMs. (A) Linear absorption: electron (blue dot) and hole (red dot) generation due to interband absorption. (B–C) Nonlinear absorption: increasing light intensity can lead to reduced absorption due to Pauli blocking (B) or increased absorption due to excited state absorption (C). (D) Photon fluence-dependent transmittance of a BP film at two orthogonal polarizations [138] © (CC-BY 4.0). (E) Optical transmittance of a BP flake as a function of photon fluence and light polarization. Reprinted figure with permission from [134] © 2015 AIP Publishing LLC. (F) Light intensity-dependent absorption coefficients of ReS_2_ crystals with distinct layer stacking orders, AA and AB [141]. Reprinted with permission from [141]. Copyright 2021 American Chemical Society.

SA describes the phenomenon where the optical absorption decreases as the intensity of light increases. In the regime of weak light intensity within the linear range, electron–hole pairs are generated through interband absorption (Figure 5A). However, at high light intensities, a large density of electron–hole pairs generated through interband absorption occupies the CB and VB, inhibiting further light absorption due to the Pauli exclusion principle. This leads to a decrease in the absorption coefficient [129]. The relationship between the absorption coefficient (*α*) and light intensity (*I*) is expressed as follows [129]

(3) $$\alpha =\frac{\alpha_{0}}{1+\left(\frac{I}{I_{s}}\right)},$$

where *α*_0_ is the linear absorption coefficient and *I*_*s*_ is the saturation intensity. This effect plays a key role in various applications such as pulse laser generation and all-optical modulators. In contrast to SA, the absorption coefficient can increase as the light intensity increases, leading to reverse saturable absorption (RSA) [130]. Excited state absorption (ESA) is one of the representative causes of RSA, where under strong irradiation, photo-excited carriers can absorb additional photons to transition to higher energy states, inducing absorption coefficient that increases with light intensity (Figure 5C). RSA can also be induced by other mechanisms, such as nonlinear scattering [131], free carrier absorption (FCA) [132], and two-photon absorption (TPA) [133], [134]. SA and RSA are identified through various techniques such as z-scan or intensity-scan [129], [130], [135], [136], [137]. We here review studies that have highlighted the anisotropic polarization-dependent nature of SA and RSA in A2DMs. Please note that there are numerous studies focusing on ultrafast pulse laser applications based on SA in A2DMs; these will be reviewed in Section 5.

Figure 5D shows the photon fluence-dependent transmittance of 1100-nm-thick BP measured using 1550 nm wavelength light [138]. As discussed earlier, greater absorption (i.e., lower transmittance) is observed in the AC polarization compared to the ZZ polarization. In both polarizations, the transmittance increases with increasing fluence, attributed to the reduction in absorption coefficient by SA. More complex polarization-dependent nonlinear absorption in BP was observed by Sotor et al. [134]. The authors measured fluence-dependent transmittance by rotating the polarization for 1560 nm wavelength light in a 300-nm-thick BP sample. In AC polarization (represented as *E*‖*x* – axis in Figure 5E), typical SA behavior is observed. In contrast, ZZ polarization (represented as *E*‖*y* – axis in Figure 5E) exhibits RSA due to TPA at high fluences above ∼200 μJ cm^−2^. Similar polarization-dependent transitions from SA to RSA have been observed in the puckered orthorhombic GeS, demonstrating polarization-controlled all-optical switching based on this anisotropic absorption nonlinearity [139]. Polarization-dependent transitions from SA to RSA have also been observed in anisotropic rhenium dichalcogenides. Meng et al. performed a polarization-dependent intensity scan on bulk ReS_2_ [130], revealing that when the light polarization is parallel to the Re-chain direction (*b*-axis), RSA is observed due to ESA of excitons polarized in that direction. However, as the polarization angle rotates, SA gradually dominates over RSA. Subsequently, the arrangements of ReS_2_ layers were shown to significantly influence polarization-dependent nonlinear absorption [140], [141], [142]. ReS_2_ exhibits two stacking modes, AA and AB, which can be distinguished by Raman spectroscopy. Zhou et al. found that SA in ReS_2_ is only possible at specific polarization angles for the AB-stacked layer, while it is absent in the AA-stacked layer (Figure 5F) [141]. These findings highlight the simultaneous importance of layer stacking order and light polarization in optical nonlinearity.

### 3.2 Harmonic generation

Harmonic generation is a nonlinear optical process in which *n* photons with the same energy interact with a material to generate a new photon with *n* times the energy [143], [144]. This phenomenon is an important mechanism used for wavelength conversion in photonics and finds extensive uses in material characterizations. In particular, harmonic generation in A2DMs exhibits unique polarization-dependent characteristics. Here, we focus on the most commonly studied SHG and THG.

**Figure 6:**
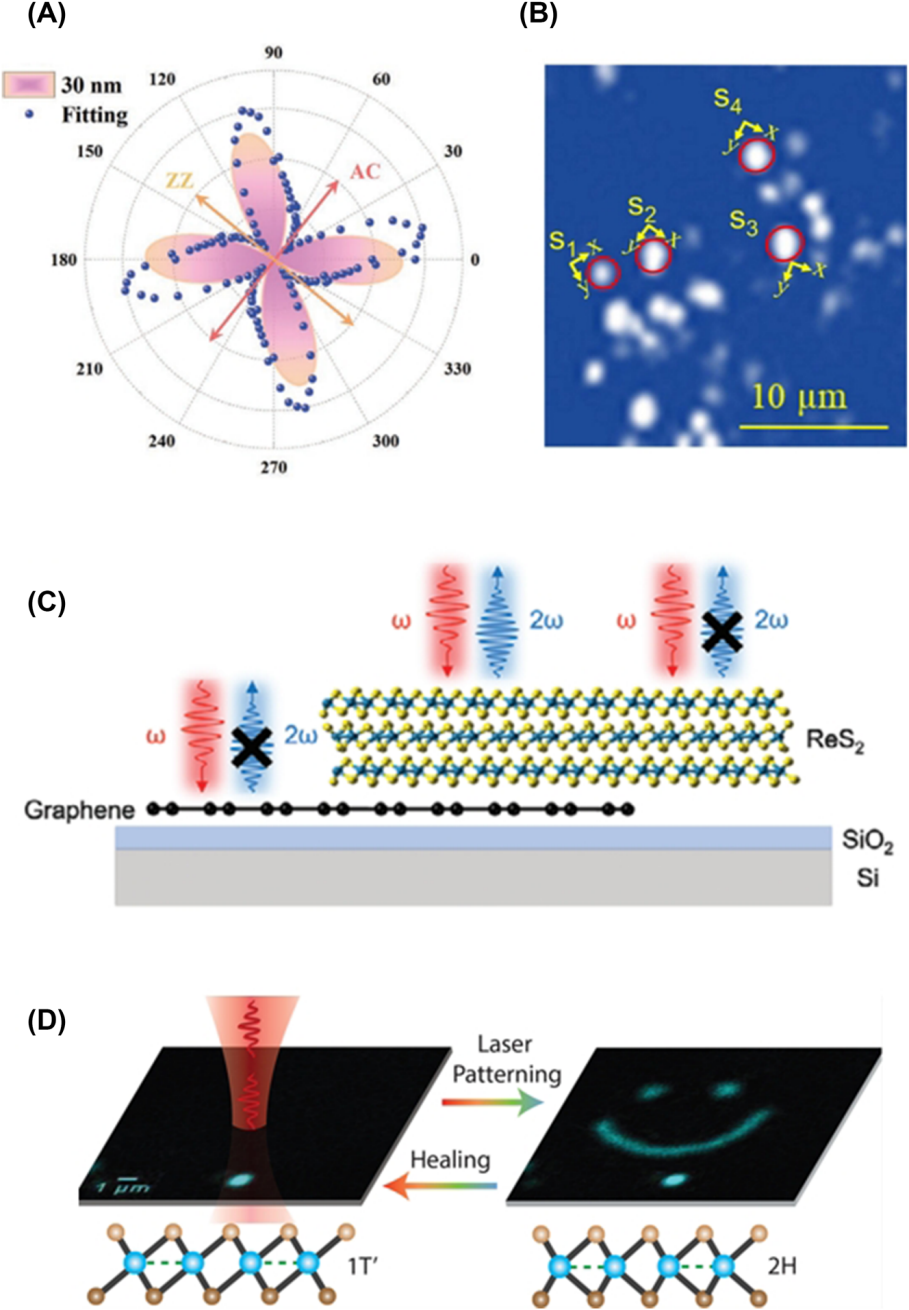
Harmonic generations in A2DMs. (A) Polarization-dependent SHG in SnS film. Reprinted figure with permission from [146] © 2021 Wiley-VCH GmbH. Reprinted figure with permission from [148] © 2022 IOP Publishing Ltd. (B) Identification of crystal orientations of few-layer SnS flakes. (C) SHG generation from ReS_2_/graphene heterostructure. Reprinted figure with permission from [151] © 2023 Wiley-VCH GmbH. (D) Laser patterning-induced SHG in monolayer ReS_2_ [153] © (CC-BY 4.0).

As SHG occurs only when inversion symmetry is broken, it is not allowed in centrosymmetric BP. However, SnS with a similar puckered orthorhombic structure as BP exhibits efficient SHG due to its broken inversion symmetry [145], [146], [147], [148]. Figure 6A shows polarization-dependent SHG measured in 30-nm-thick SnS, revealing a four-fold pattern governed by two-fold rotational symmetry [146]. Leveraging this characteristic pattern, polarization-dependent SHG imaging has been successfully employed to identify the crystal axes of multiple SnS flakes (Figure 6B) [147], [148].

Anisotropic SHG in 2D ReS_2_ has also been extensively investigated. Notably, the occurrence of SHG depending on the layer number parity has been a crucial issue. Odd-layer ReS_2_ does not allow SHG due to centrosymmetry, whereas even-layer ReS_2_ exhibits SHG due to the broken symmetry [149]. Recently, Song et al. revealed that only certain structures with specific stacking orders among even-layered ReS_2_ allow SHG [150]. It has been demonstrated that SHG can be induced in odd-layer ReS_2_ through external manipulations such as hetero-stacking [151], gate biasing [152], and laser patterning [153]. Wang et al. showed that SHG occurs at the heterostructure of odd-layer ReS_2_ and graphene (Figure 6C), attributed to the breaking of centrosymmetry by charge transfer at the ReS_2_/graphene interface [151]. Küçüköz et al. induced a transition from the centrosymmetric 1T′-phase to the noncentrosymmetric 2H-phase in monolayer ReS_2_ using laser patterning, significantly enhancing SHG intensity (Figure 6D) [153]. In addition to binary materials like SnS and ReS_2_, efficient anisotropic SHG has also been observed in the non-centrosymmetric ternary compound Nb_3_SeI_7_ [118].

THG is allowed regardless of the structural centrosymmetry. Thus, it has been extensively observed in centrosymmetric materials such as BP and monolayer ReS_2_, exhibiting characteristic polarization dependences [154], [155], [156], [157]. In BP, the polarization-dependent nature of THG has been utilized for crystal orientation determination through wide-area scanning [156]. Furthermore, the THG efficiency has been found to exhibit a high thickness dependence due to phase matching conditions and depletion within the flake [155].

## 4 Anisotropic ultrafast optical properties

To explore the ultrafast dynamics in 2D materials in a time-resolved manner, various experimental methods have been employed, including transient absorption (TA), time-resolved photoluminescence (TRPL) [6], ultrafast THz spectroscopy [158], [159], and time-resolved angle-resolved photoemission spectroscopy (TR-ARPES) [160]. All of these techniques serve as powerful tools for revealing aspects of photo-induced dynamics and light-matter interactions. TRPL can detect the ultrafast radiative dynamics of photoexcited carriers, while ultrafast THz spectroscopy enables the direct observation of charge carrier dynamics and transitions between excitons’ Rydberg states [158], [159], [161], [162], [163]. TR-ARPES captures the ultrafast behavior of carriers in energy-momentum space, providing direct insights into carrier scattering, transfer, and relaxation [160]. Among these various time-resolved experimental tools, TA has been widely utilized for investigating the anisotropic polarization-dependent ultrafast photoinduced dynamics in A2DMs. Unlike TRPL, TA allows direct observation of the dynamics of non-radiative carriers, and it has a relatively simpler experimental setup compared to TR-ARPES. Additionally, TA can be easily integrated with microscopy. In contrast, THz spectroscopy relies on terahertz wave lengths, resulting in a diffraction-limited resolution of a few hundred micrometers. Therefore, measuring small 2D single-crystal flakes with sizes in the range of tens to hundreds of micrometers poses some challenges. Although various THz microscopy techniques have been developed recently [164], they have not been as widely employed for studying anisotropic dynamics in A2DMs as TA-based microscopy. Hence, we here primarily focus on exploring the ultrafast anisotropic dynamics of A2DMs using TA.

**Figure 7:**
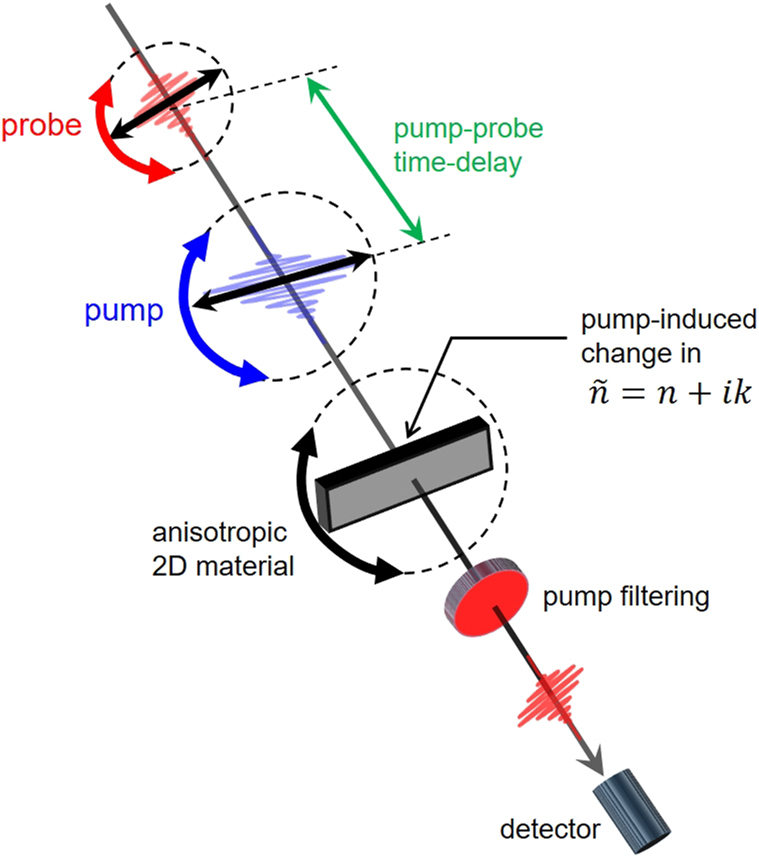
Schematic of TA experiment.

TA is a pump-probe type of time-resolved spectroscopy primarily utilizing femtosecond pulse laser sources. Figure 7 illustrates a simplified experimental setup for TA. Typically, the energy of the pump photons is set above the bandgap or at the absorption resonances of the sample material, except in intentional cases such as optical Stark effect (OSE) experiments where below-gap excitation can be used [165], [166], [167]. The probe pulse is configured to have a relatively weaker intensity compared to the pump pulse. Upon pump excitation, the optical characteristics of the sample undergo modulation, causing a change in the intensity of the probe beam that either passes through the sample or reflects from it. When detecting the transmitted probe beam, as shown in Figure 7, the signal is often recorded as $\frac{T_{\text{pump}}-T_{0}}{T_{0}}=\frac{\Delta T}{T_{0}}$ , where *T*_pump_ and *T*_0_ denote the probe intensity detected by the detector when the pump is turned on and off, respectively. $\frac{\Delta T}{T_{0}}$ is also referred as differential transmission. When detecting the probe beam reflected from the sample, the measured signal is the differential reflection, defined as $\frac{R_{\text{pump}}-R_{0}}{R_{0}}=\frac{\Delta R}{R_{0}}$ . Here, *R*_pump_ (*R*_0_) represents the intensity of the probe reflected when the pump is present (absent). The time delay between the pump pulse and the probe pulse can be changed using a mechanical delay stage, enabling the recording of TA signals in the form of $\frac{\Delta T}{T_{0}}$ and/or $\frac{\Delta R}{R_{0}}$ as functions of time.

In polarization-dependent TA, wave plates can be employed to rotate the polarization of the pump beam (round blue arrow in Figure 7) or the probe beam (round red arrow in Figure 7). Alternatively, a rotation stage can be used to rotate the sample while keeping both the pump and probe polarizations fixed (round black arrow in Figure 7). These approaches reveal the anisotropy of TA with distinct patterns and physical origins, which will be discussed below.

### 4.1 Anisotropic carrier dynamics

#### 4.1.1 Principle of TA signal generation by pump-excited carriers

The optical properties of a material can be expressed using the complex refractive index ( $\tilde{n}$ ), denoted as [3]:

(4) $$\tilde{n}=n+ik.$$

Here, the real part *n* on the right-hand side represents the refractive index, and the imaginary part *k* is the extinction coefficient. The latter is directly related to the absorption coefficient by the following relation [168]:

(5) α=4πk/λ,

where *λ* is the wavelength of light. In TA experiments, as the pump excites the sample, the complex refractive index of the sample is transiently modulated. This modulation affects the intensity of the probe beam passing through the sample and/or reflected from the sample, giving rise to differential transmission and differential reflection signals, respectively. Here, both the real and imaginary parts of the complex refractive index can contribute to the TA signal due to pump-induced changes. However, resolving their individual contributions requires rigorous analysis [169], [170], [171]. In many cases, the interpretation of TA signals is based on changes in the extinction coefficient induced by the pump, which corresponds to variations in the sample’s absorption properties. In this context, pump-induced absorption changes in the probe beam can either decrease or increase, referred to as photo-bleaching (PB) and photo-induced absorption (PA), respectively. PB corresponds to the SA discussed in Section 3, where pump light leads to reduced absorption. Conversely, PA corresponds to RSA, as it results in increased absorption due to the pump.

**Figure 8:**
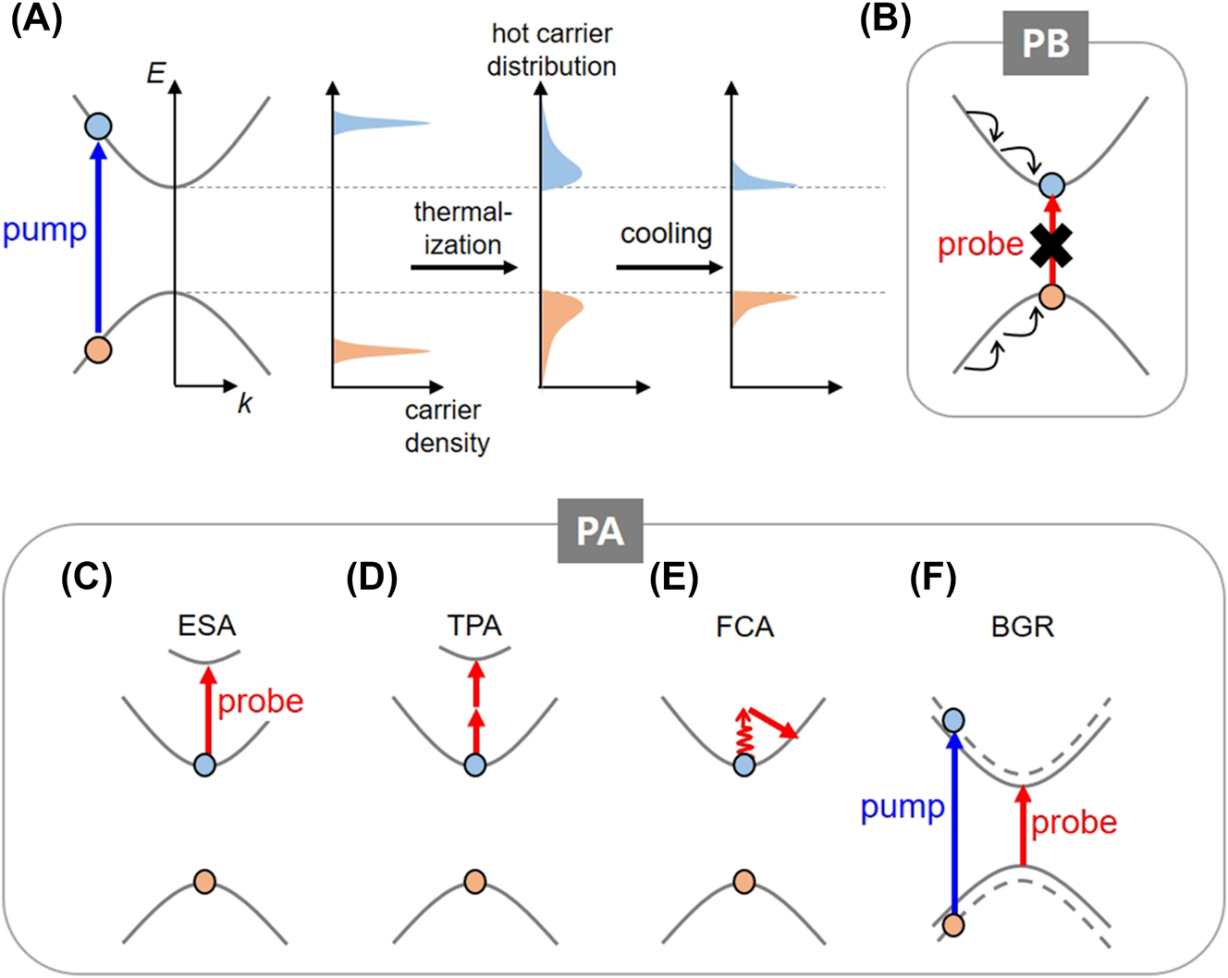
Various causes for TA signals. (A) Illustration of carrier dynamics under pump excitation. (B) Photo-bleaching (PB): state filling by pump-generated carriers induces Pauli blocking of the probe absorption. (C–F) Photo-induced absorption (PA) mechanisms: excited-state absorption (ESA), two-photon absorption (TPA), free-carrier absorption (FCA), and bandgap renormalization (BGR).

To understand the origins of PB and PA, let us first examine the processes that occur immediately after pump excitation. When electrons and holes with excess energy are generated through interband absorption of pump photons, they undergo rapid carrier–carrier scattering and thermalize within hundreds of femtoseconds [172]. As a result, a quasi-Fermi distribution forms, defining the carrier temperature. Carrier thermalization establishes the hot carrier distribution, and then hot carriers cool down to band edges through interactions with phonons. The carrier cooling time scales 2D semiconductors typically span from tens of femtoseconds to picoseconds, depending on the material type, thickness, and pump intensity [102], [173], [174], [175], [176]. Carrier cooling is generally delayed by the excess carrier energy, which increases with higher pump energy [177], while defects can accelerate the cooling process [178]. The initial carrier thermalization and cooling processes are depicted in Figure 8A.

Next, we consider a case where the probe energy is set to coincide with an interband transition from the CB edge to the VB edge, as indicated by the red arrow in Figure 8B. The cooled carriers now fill the CB and VB edge states, causing a blockage in the absorption of probe photons due to the Pauli exclusion principle. This results in decreased absorption (i.e., PB). Naturally, PB at different probe energies can arise due to state filling as well. PB can also be triggered by other processes such as line-shape modulations, which will be discussed later with specific examples.

PA originates from a diverse set of mechanisms, including ESA, TPA, FCA, and bandgap renormalization (BGR). ESA involves the excitation of pump-generated carriers, causing them to absorb probe photons and transition to higher-energy states (Figure 8C), displaying peak-like [179], [180] or broad spectral features [181], [182]. These excited-state transitions can even result from absorbing two probe photons (Figure 8D) [183]. FCA emerges from the interaction between pump-created carriers and probe photons, giving rise to intraband transitions (Figure 8E). While commonly explained through the Drude model [184], [185], recent findings highlight inverse bremsstrahlung-type FCA due to carrier interactions with photoinonized ions [186]. FCA tends to intensify with longer photon wavelengths, making it frequently observable through low-energy probes like THz or mid-infrared [158], [159], [185], [187], [188], [189]. Nevertheless, it is also observable in the NIR-visible region [190], [191], [192]. BGR refers to the contraction of the band gap, driven by screened repulsive Coulomb interactions caused by pump-generated carriers [193], [194], [195], [196], [197]. Particularly in 2D materials, poor dielectric screening makes Coulomb interactions highly sensitive to pump-generated carriers. Hence, 2D materials often exhibit stronger BGR compared to their bulk counterparts [198]. The band-gap reduction due to BGR allows for new interband transitions below the original bandgap, resulting in PA (Figure 8F). However, in many cases, multiple effects coexist with BGR. For instance, the relaxation of carriers filling band-edge states leads to PB, which can mask the PA signal induced by BGR [199].

#### 4.1.2 Decay dynamics

The explained PB and PA originate from carriers generated by the pump. The carrier concentration diminishes over time due to various mechanisms such as carrier trapping by defects, non-radiative recombination, phonon-assisted recombination, and radiative recombination [6], [200], [201]. Resultantly, TA signals decay with pump-probe time-delay. The corresponding decay traces provide information on underlying carrier dynamics mechanisms and operational time scales for related devices. In this section, we briefly review prominent carrier depopulation mechanisms commonly observed in 2D materials.

Due to their high surface-to-volume ratio, 2D materials are susceptible to environmental influences and prone to the formation of defects [200]. These defects trap carriers generated by pump, inducing depopulation. Various time scales of defect-related depopulation, ranging from sub-picoseconds to hundreds of picoseconds, have been observed in different 2D materials [200]. Even within a single material, the types of defects can lead to the emergence various energy levels, each causing distinct carrier trapping times [202], [203]. For instance, in ReS_2_, sulfur vacancies dominate as defects, manifesting as shallow and deep energy levels within the bandgap [204]. Wang et al. observed that in ReS_2_ thin films, most photo-generated carriers are trapped within the shallow-level defect within 1 ps, while a smaller portion of carriers undergo a slower process associated with the deep defect level [203]. One notable signature of carrier trapping by defects is the increase in carrier lifetime as pump intensity rises [205], [206]. Due to the limited number of defect sites, defect sites can be saturated by trapped carriers, hindering additional carrier trapping and causing delayed decay. However, this trend may not hold true when the amount of pump-generated carriers is significantly lower or higher than the defect concentration [207]. For a more comprehensive discussion of defect-related carrier dynamics in 2D materials, including isotropic materials, recent excellent review papers are available for reference [200].

Many-body Auger recombination is another frequently observed depopulation process in 2D materials [203], [205], [208], [209], [210]. In the conventional three-body Auger recombination involving free carriers, one electron and one hole non-radiatively recombine, exciting another electron (or hole) to a higher state. As the Auger process emerges from carrier interactions, it becomes more pronounced with elevated carrier density. Consequently, the Auger recombination-induced decay of TA signals tends to accelerate as the pump intensity increases [205], [208], [211]. This pump intensity-dependent trend is contrary to the trapping by defects mentioned earlier. Defect-assisted Auger recombination is also observed in 2D materials [203], [205], [209], [210], [212]. In this process, carriers trapped by defects interact with free carriers, facilitating rapid depopulation. While this process is inherently a three-body mechanism, under specific conditions, it can also be described by a two-body bimolecular model [205], [212], [213].

In narrow bandgap materials, electrons and holes can directly recombine by emitting bosons, such as phonons or magnons. However, the emitted bosons can re-excite the electrons and holes across the narrow bandgap, leading to a delayed decay [214]. These recombination processes are described by the Rothwarf–Taylor (RT) model and have been observed in anisotropic narrow-gap materials such as ZrTe_5_ [180], [215], [216].

#### 4.1.3 Examples of anisotropic carrier dynamics in A2DMs

**Figure 9:**
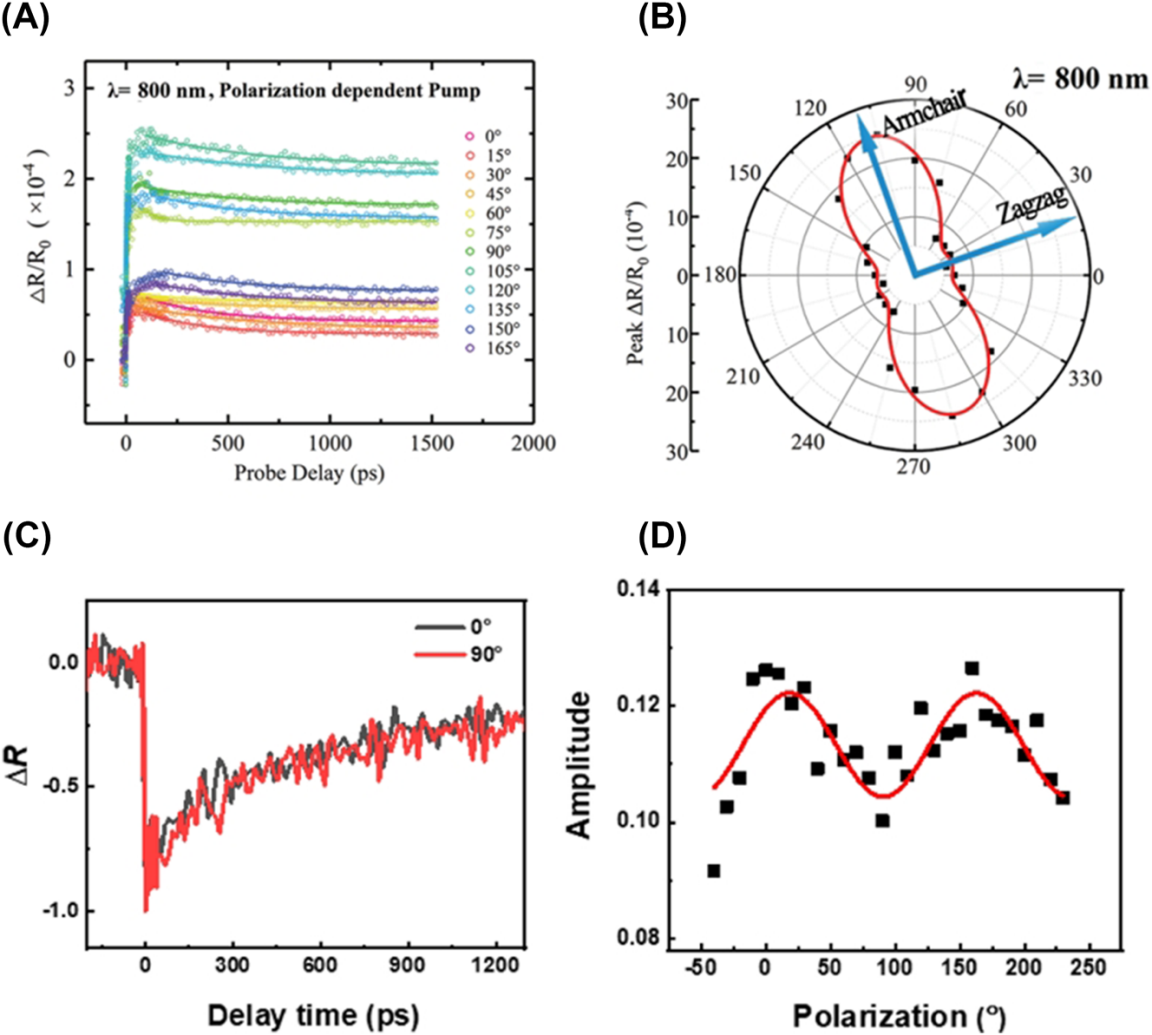
Pump polarization-dependent anisotropic carrier dynamics. (A–B) Differential reflection traces measured from SnSe at different pump polarizations (A) and corresponding peak values (B). Reprinted figure with permission from [217] © 2019 WILEY-VCH Verlag GmbH & Co. KGaA, Weinheim. (C–D) Differential reflection signals in NbTe_2_ at two perpendicular pump polarizations (C) and absolute peak values of differential reflection as a function of pump polarization (D). Reprinted with permission from [218]. Copyright 2022 American Chemical Society.

We above mentioned various methods for investigating the anisotropy of A2DMs using TA, including pump polarization rotation, probe polarization rotation, and sample rotation (Figure 7). We first examine cases of pump polarization-dependent TA. Figure 9A displays the differential reflection traces measured while rotating pump polarization, taken with a 400-nm pump and 800-nm probe on a 175 nm-thick SnSe flake [217]. As a result of state filling by pump-generated carriers, probe absorption decreases (i.e., PB), leading to an increase in reflection signal $\left(\frac{\Delta R}{R_{0}}>0\right)$ . The peak $\frac{\Delta R}{R_{0}}$ is maximized in AC polarization and is about 4.6 times larger than that in ZZ polarization (Figure 9B). This anisotropic response is attributed to relatively higher absorption in the AC direction of pump polarization, resulting in the generation of more carriers and a more pronounced PB effect. Conversely, the decay time of TA traces, ascribed to carrier relaxation time, remains almost unchanged with polarization. As another example, Figure 9C presents normalized traces of pump polarization-dependent differential reflection measured on 32-nm-thick anisotropic NbTe_2_ on a quartz substrate [218]. These signals exhibit PB, but unlike Figure 9A, they are negative in sign. This is due to the fact that for thin samples on a transparent substrate, the change in reflection can be proportional to the change in absorption. While NbTe_2_ shows pump absorption anisotropy (Figure 9D), the normalized dynamics exhibit almost no polarization dependence (Figure 9C). Such trends of pump polarization dependence have also been observed in other A2DMs, including ZrTe_5_ [180] and TiS_3_ [102]. The weak pump polarization dependence in normalized carrier dynamics is attributed to the fast disappearance of pump polarization-induced memory due to rapid carrier-carrier scattering shortly after pump excitation, within the typical time resolution of TA experiments (∼10 s–100 s fs) [180], [219].

**Figure 10:**
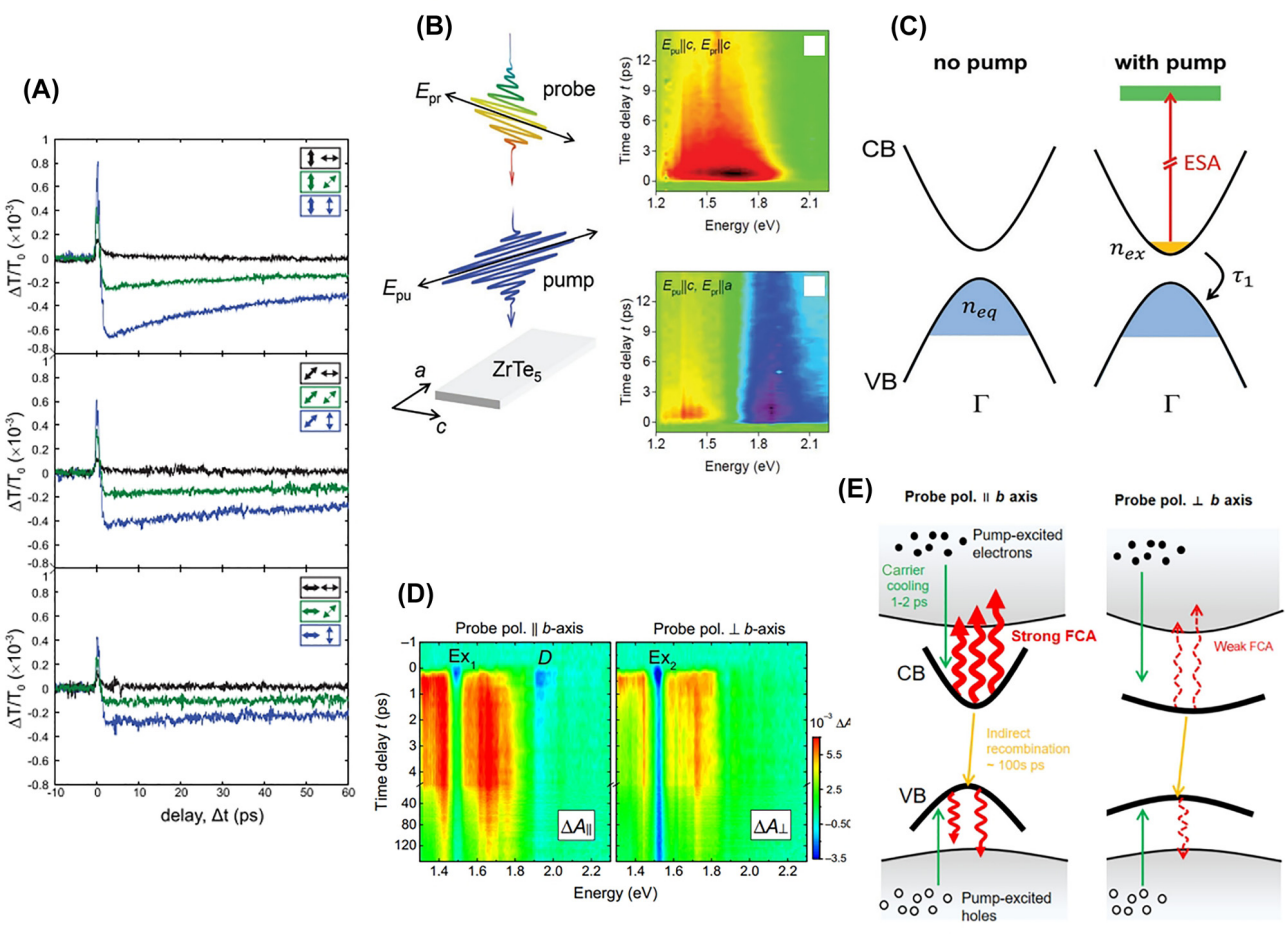
Probe polarization-dependent anisotropic carrier dynamics. (A) Differential transmission dynamics measured from ∼80-layered BP. The arrows on the left and right denote the polarization of the pump and probe, respectively. Reprinted figure with permission from [220] © 2015 AIP Publishing LLC. (B–C) Schematic of the TA experiment on ZrTe_5_ crystal and resulting differential transmission maps for probe polarizations along the crystal *c*-axis (upper right) and the *a*-axis (lower right). (C) Indicates that photo-induced absorption (PA) with *c*-axis probe polarization is attributed to excited-state absorption of pump-generated electrons. Reprinted figure with permission from [180] © 2022 Wiley-VCH GmbH. (D–E) Absorption change maps of few-layer ReS_2_ under 400-nm pump excitation (D). The panels on the left and right in (D) represent data obtained when the probe polarization is parallel and perpendicular to the crystal *b*-axis, respectively. A schematic illustrates that relatively strong (weak) free carrier absorption occurs with polarization parallel (perpendicular) to the *b*-axis, resulting in anisotropic PA. Reprinted figure with permission from [192]. Copyright 2022 by the American Physical Society.

Many studies on A2DMs have investigated both pump- and probe-polarization-dependent dynamics, but significant changes in dynamics have often been observed in the latter case [102], [122], [180], [192], [219], [220]. Suess et al. conducted differential transmission experiments on multilayer BP with 780-nm-pump and 1560-nm-probe [220]. When the probe polarization is in the horizontal direction (black curves in Figure 10A), only positive transmission changes (i.e., PB) are observed, while in the vertical probe polarization (blue curves in Figure 10A), the initial peak-like PB response is followed by a prolonged PA due to Drude absorption. In contrast, comparing the three panels of Figure 10A, it is evident that changes in pump polarization primarily affect the overall scale of the TA signal and have minimal impact on the time-dependent dynamics, similar to the cases of SnSe and NbTe_2_ discussed above.

To comprehensively explore the probe polarization dependence in a spectrally-resolved manner, broadband probe-based TA experiments have also been conducted. Seo et al. observed highly anisotropic probe polarization dependence for ZrTe_5_ nanoribbons [180]. The ribbons exhibit an elongated shape along the crystal *a*-axis due to quasi-1D Zr–Te chains, with the *c*-axis being perpendicular to the *a*-axis. The authors observed significant probe polarization dependence in the range of 1.2–2.2 eV, as shown in Figure 10B. Particularly, strong PB was observed at 1.62 eV when the probe polarization is along the *c*-axis, and interestingly, it diminishes as the probe polarization rotates to the *a*-axis. Furthermore, this PA exhibited a fast decay of about 2 ps, consistent with the RT-model description. Such ultrafast and complete anisotropy offers promising potential for polarization-driven optical modulation. The anisotropic PA is attributed to *c*-axis-polarized ESA through calculations of transition dipole moments (Figure 10C). Seo et al. also investigated the anisotropic behavior of few-layer ReS_2_ using broadband probe [192]. Figure 10D displays the TA maps at two orthogonal probe polarizations under a 400-nm pump. The pronounced broad PA feature highlighted in red is more prominent with the *b*-axis polarization (left panel), while it appears comparatively weaker with the perpendicular polarization (right panel). The authors attributed these anisotropic broadband PA features to FCA. In ReS_2_, the effective masses of band-edge carriers are smaller in the *b*-axis direction compared to does along the perpendicular direction, as illustrated by the band curvatures in Figure 10E. Since FCA coefficients are negatively correlated with carrier effective masses, a relatively larger FCA is induced in the *b*-axis direction, resulting in the stronger PA feature. This study exemplifies cases where direction-dependent carrier effective masses can lead to anisotropic behavior in broadband transient photoresponses.

**Figure 11:**
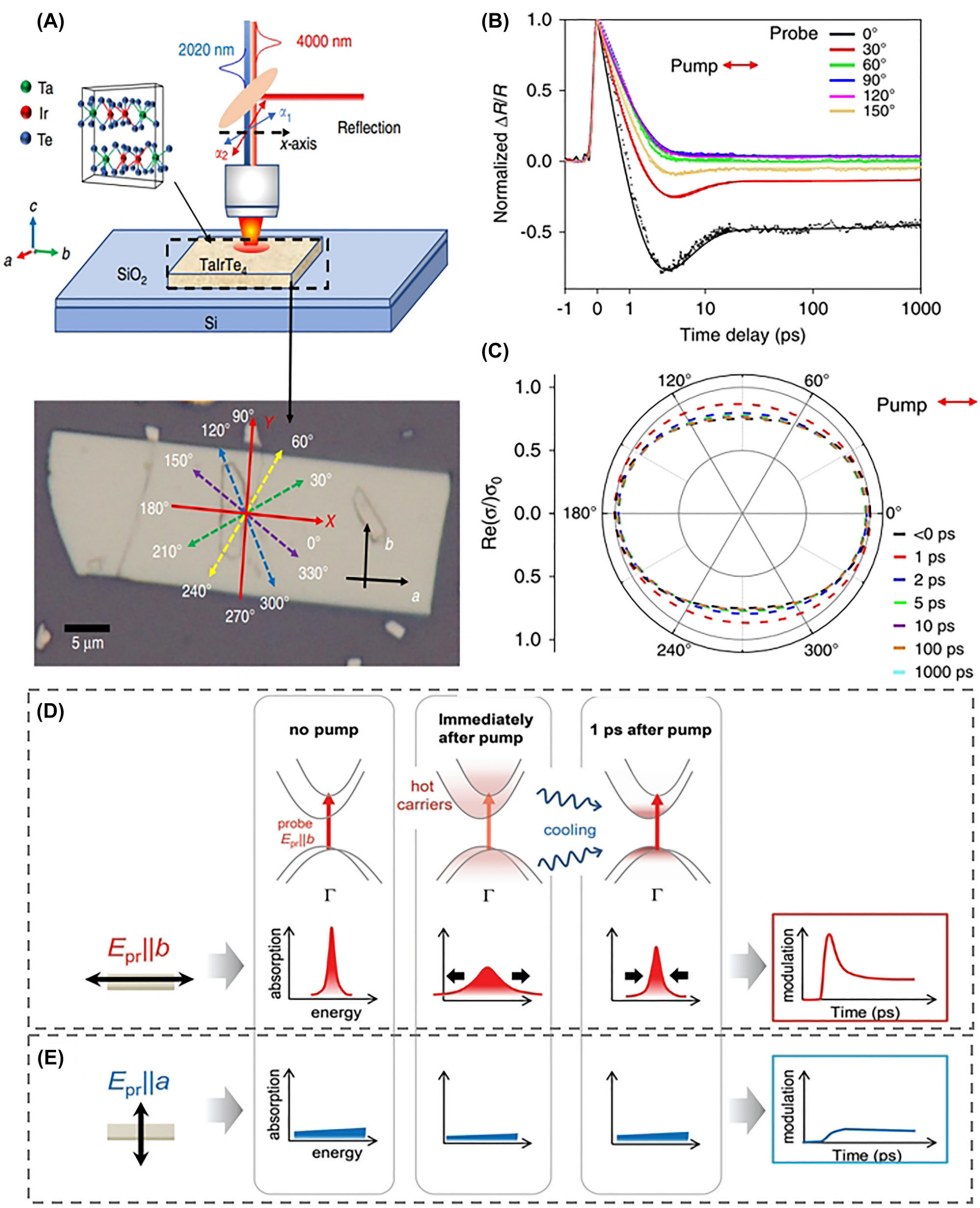
Hot carrier-induced ultrafast anisotropic response. (A–C) Schematic of polarization-dependent differential reflection experiment on TaIrTe_4_ (A). Dynamics observed while varying probe polarizations (B) and hot carrier-induced ultrafast changes in direction-dependent optical conductivity (C) [122] © (CC-BY 4.0). (D–E) Summary of polarization-dependent TA responses measured in TiS_3_. When the probe polarization is perpendicular to the *b*-axis, peak broadening due to pump-generated hot carriers and subsequent ultrafast decay resulting from their cooling is observed (D). In contrast, such ultrafast responses are not observed when the probe polarization is perpendicular to the *b*-axis. Reprinted figure with permission from [102] © 2023 Wiley-VCH GmbH.

Interesting anisotropic TA responses driven by hot carriers have also been observed. Zhuo et al. performed polarization-dependent MIR differential reflection measurements on TaIrTe_4_, known as a type-II Weyl semimetal (Figure 11A) [122]. They observed that, near zero pump-probe time delay, all differential reflection traces exhibit positive peaks, while later on, the sign of the signal changes depending on the probe polarization (Figure 11B). The authors identified that the differential reflection signal originates from hot carriers, and it is proportional to the change in the real part of the complex conductivity. Thus, in Figure 11B, the negative $\frac{\Delta R}{R_{0}}$ observed at *x*-axis (0°) indicates a decrease in conductivity, while the positive $\frac{\Delta R}{R_{0}}$ at *y*-axis (90°) indicates an increased conductivity. The total response leads to the dynamic decrease of conductivity anisotropy (Figure 11C). Interestingly, this finding contrasts with the result in BP, where the anisotropy of transient conductivity is increased by hot carriers [219]. Suk et al. observed anisotropic hot carrier dynamics using differential transmission for quasi-1D TiS_3_ nanoribbons [102]. When the probe polarization is parallel to the long axis of the ribbon (*b*-axis), an interband absorption peak near 1.3 eV undergoes instantaneous broadening due to hot carriers and rapidly returns through cooling, leading to a sub-picosecond PB dynamics (Figure 11D). On the other hand, when the probe polarization is perpendicular to the *b*-axis, the interband absorption peak disappears, and consequently, the ultrafast sub-picosecond TA component also vanishes (Figure 11E). These results provide insights into the operating principles of ultrafast modulation controlled by polarization.

Beyond rotating the pump and probe polarizations, sample rotation has also been widely used [221], [222], [223]. By rotating the sample, the relative angles between specific crystal axes and both the pump and probe polarizations are altered. Consequently, the anisotropy influenced by both pump and probe polarizations simultaneously affects TA signals, which can lead to intricate angle-dependent patterns. An illustrative case is ReSe_2_ [222], where a distinctive four-fold TA pattern as a function of sample angle has been observed, setting it apart from the two-fold patterns mostly observed in other A2DMs.

### 4.2 Anisotropic exciton dynamics

**Figure 12:**
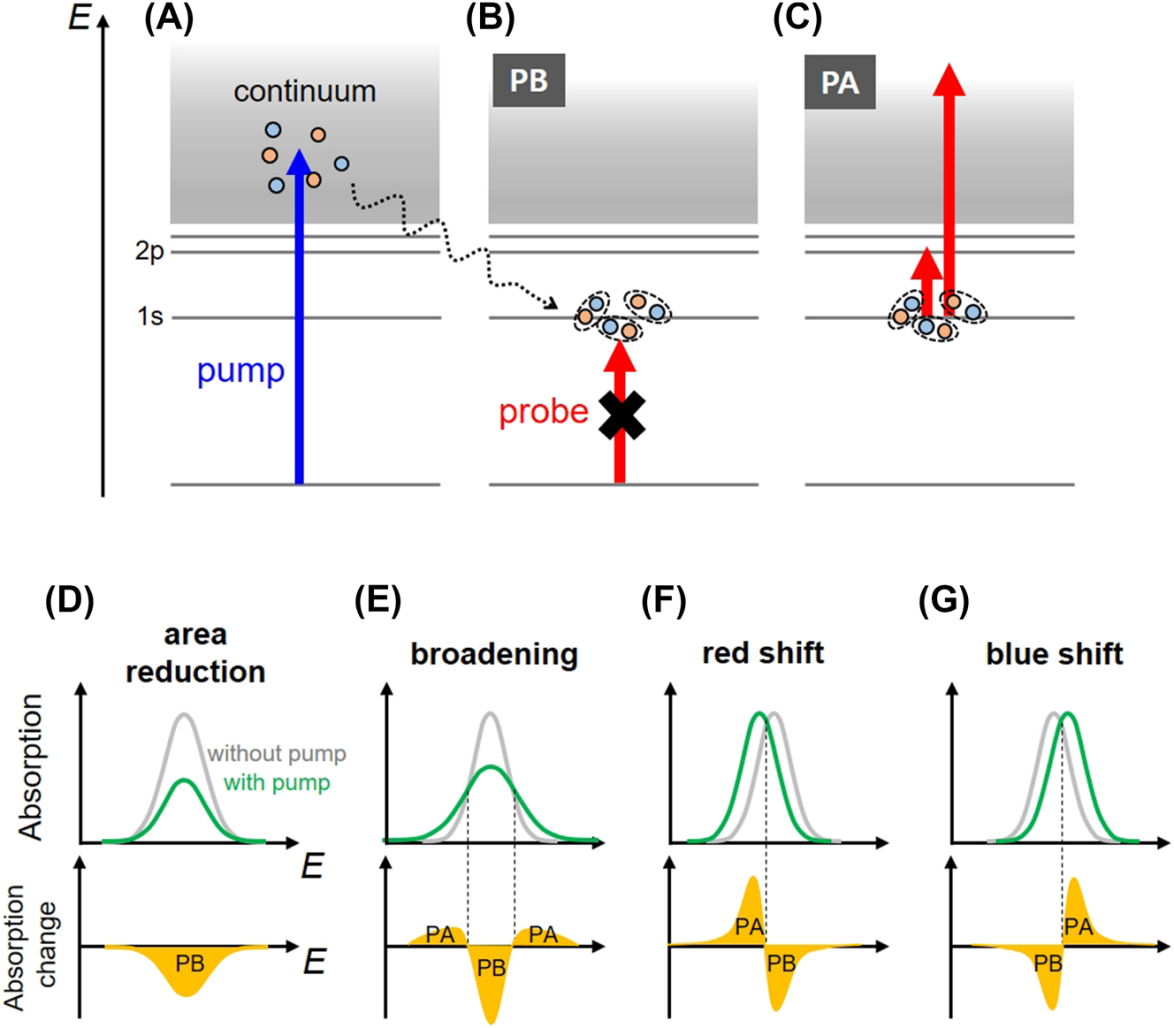
Exciton dynamics in 2D semiconductors. (A–C) Non-resonant pump excitation generates free carriers (A), followed by thermalization, cooling, and exciton formation. These excitons, through phase-space filling, can cause a decrease in probe absorption, i.e., PB (B) or induce PA signals through ESA (C). (D–G) Gray curves represent unperturbed exciton absorption resonances, while green curves depict resonances modulated by pump excitation. The difference between these, i.e., absorption change, is shown as the yellow graphs. The changes in exciton line-shape can occur in various ways, including area reduction (D), broadening (E), red-shift (F), and blue-shift (G).

Excitons in 2D semiconductors not only determine their linear optical properties but also significantly influence ultrafast optical phenomena. In TA experiments, excitons can be directly formed by tuning the pump photon energy to match the exciton resonance. However, in order to avoid pump scattering to the detector or to induce strong pump absorption, excitations with energies higher than the pre-particle bandgap are often employed. Under such non-resonant, above-gap excitations (Figure 12A), free carriers are initially generated and undergo carrier-carrier scattering to thermalize within a few hundred femtoseconds. Subsequently, carrier relaxation to lower energies occurs through cooling processes such as electron-phonon scattering. Up to this point, the processes are identical to those described in Figure 8A. In 2D semiconductors, a large portion of free carriers can form excitons even at room temperature due to their large exciton binding energies, unless the exciton density exceeds the Mott density [224]. The dotted wavy arrow in Figure 12A and B illustrate this exciton formation, with corresponding time scales usually within 1 ps in 2D semiconductors [163], [225], [226].

The pump-generated exciton can give rise to both PB and PA signals through various mechanisms. When the probe energy resonates with the exciton, the absorption of the probe beam is hindered by the phase-space filling due to the electrons and holes constituting the exciton, leading to PB (Figure 12B) [227]. Alternatively, as shown in Figure 12C, excitons can absorb probe photons and transition to higher states, inducing ESA-type PA [228]. Specifically, intraexcitonic transitions where excitons jump to upper-lying Rydberg states are observable through low energy probes such as THz or MIR, providing crucial information about exciton structures [161], [163].

The spectral line-shape variations of excitons also give rise to various forms of PB and PA signals. Firstly, phase-space filling and Coulomb screening by pump-generated electrons and holes reduce the exciton transition probability. This diminishes the area of the exciton absorption peak, leading to a negative absorption change, i.e., PB (Figure 12D) [227], [229]. Secondly, excitons generated during the probe absorption can scatter with pump-generated carriers and excitons. This leads to a reduction in the phase coherence time of excitons, resulting in line-shape broadening [196], [229], [230]. The corresponding absorption change spectrum resembles the second derivative of the absorption spectrum, exhibiting PB at the center and PA peaks at the sides (PA). Notably, although pump excitation typically induces exciton broadening, recent observations have indicated an anomalous line-shape narrowing in liquid-phase exfoliated ReS_2_ [231]. Thirdly, the exciton center energy can be red-shifted by the pump. The resulting absorption change resembles the first derivative of the absorption spectrum, showing PA on the lower energy side and PB on the higher energy side (Figure 12F). Conversely, blue-shift results in a flipped absorption change spectrum (Figure 12G). The red-shift of exciton can be caused by various factors including reduction of the bandgap due to BGR and attractive interactions between excitons [194], [196], [227], [232]. Additionally, electronic-phonon scattering can transfer electronic energy to the lattice, leading to a decrease in the bandgap and inducing exciton red-shift [196], [227]. There are also diverse causes for blue-shift. Pump-generated carriers screening Coulomb interactions reduce exciton binding energy, causing exciton blue-shift [194], [196], [227], [229], [232]. Alternatively, pump-generated carriers filling the band-edge state increase the optical bandgap, resulting in a blue-shift (Burstein–Moss effect) [233]. These various red-shift and blue-shift mechanisms compete in the time domain, showing dynamic peak-shifting behaviors [227]. Furthermore, the OSE induces transient red or blue shifts in excitons [165], [166], [167]. In this process, during the pump pulse passes through the material, transient photon-dressed virtual states can be formed, and the interaction between these states and bare excitons leads to shifts in the exciton energy.

**Figure 13:**
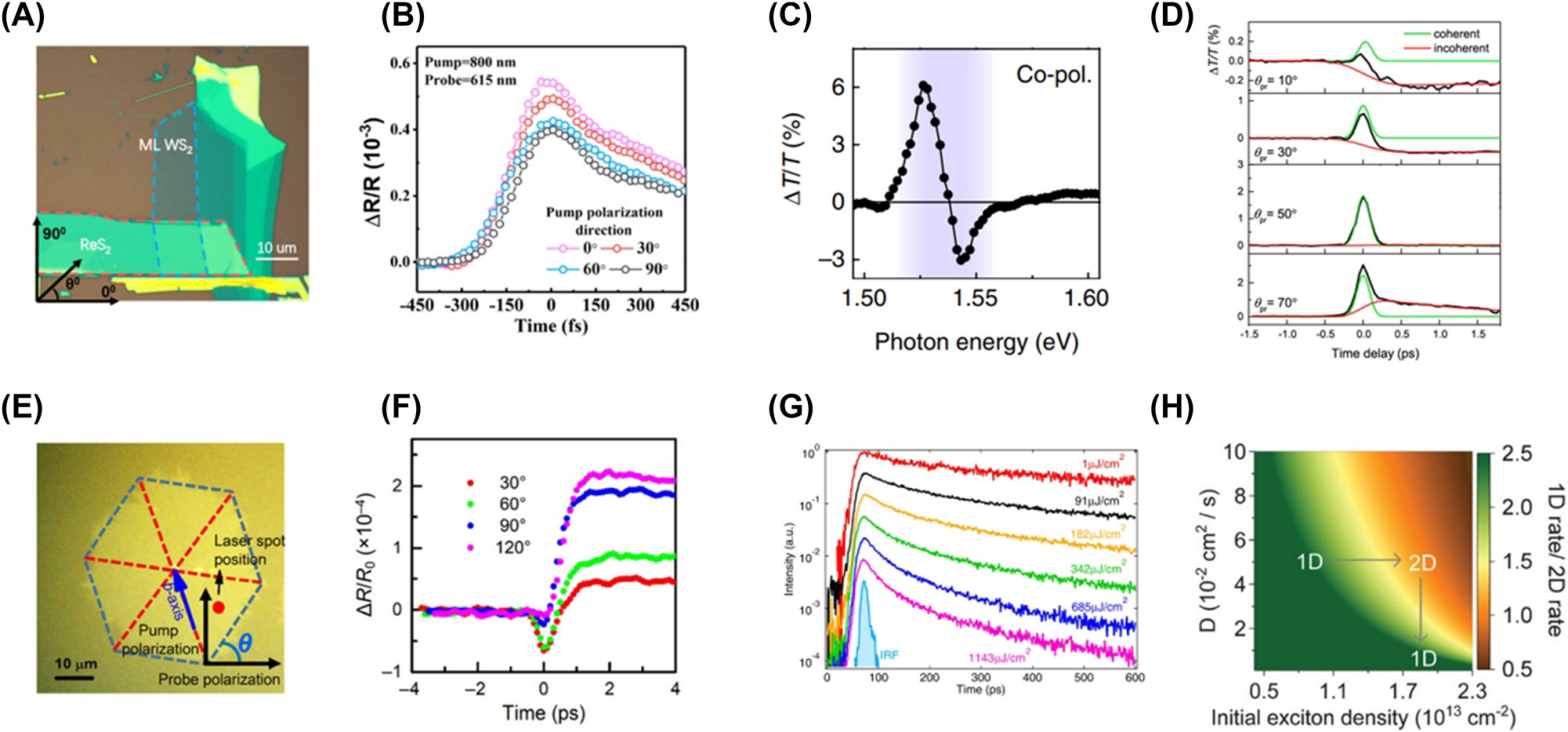
Exciton dynamics in A2DMs. (A–B) Optical image (A) and pump polarization-dependent differential reflection traces of the ReS_2_/WS_2_ heterostructure (B). An 800-nm pump was used to selectively excite ReS_2_, and a 615-nm probe was set to the A-exciton resonance of WS_2_ [235]. Reprinted with permission from [235]. Copyright 2020 American Chemical Society. (C) Transient differential transmission spectrum arising from the exciton blue-shift due to the optical Stark effect (OSE) in ReS_2_ [167] © (CC-BY 4.0). (D) Probe polarization-dependent OSE dynamics (green curves) superimposed on incoherent slow responses (red curves), measured in few-layer ReS_2_. Reprinted with permission from [237]. Copyright 2019 American Chemical Society. © 2019 American Chemical Society. (E–F) Optical image of a monolayer ReSe_2_ crystal (E) and its differential reflection signals measured while varying the sample orientation. Reprinted with permission from [239] © 2020, Tsinghua University Press and Springer-Verlag GmbH Germany, part of Springer Nature. (G) Pump fluence-dependent TRPL dynamics measured from monolayer BP. Reprinted figure with permission from [245]. Copyright 2016 by the American Physical Society. (H) Dimensionality variation of EEA in BP as a function of exciton density and diffusion constant. Reprinted figure with permission from [246]. Copyright 2020 by the American Physical Society.

With this understanding, we proceed to examine the anisotropic exciton dynamics in A2DMs. As illustrated in Figure 7, various forms of anisotropic investigations, such as pump polarization rotation, probe polarization rotation, and sample rotation, have been conducted. Firstly, pump-polarization-dependent anisotropic exciton dynamics have primarily been studied in ReS_2_ and ReS_2_-based heterostructures [234], [235], [236]. Huo et al. demonstrated the pump-polarization-dependent excitonic PA response due to the anisotropy of pump absorption in ReS_2_ [236]. Tang et al. established pump-polarization dependence in type-2 heterostructures consisting of anisotropic multi-layer ReS_2_ and isotropic monolayer WS_2_ (Figure 13A) [235]. They selectively excited ReS_2_ having a smaller bandgap using an 800-nm pump. The type-2 band alignment induced ultrafast charge transfer from ReS_2_ to WS_2_, resulting in bleaching of WS_2_’s A-exciton resonance at 615 nm. This PB signal exhibited a pronounced pump-polarization dependence (Figure 13B), driven by the anisotropy of pump absorption in ReS_2_. Based on this anisotropy and ultrafast charge transfer, the authors demonstrated high-speed polarization-selective photodetectors. Sim et al. modulated the exciton energy of few-layer ReS_2_ using OSE [167]. They set the probe polarization to match the anisotropic exciton and investigated pump polarization-dependent OSE. The pump photon energy was 90 meV lower than the exciton energy, to induce virtual photon-dressed states and to avoid generation of real excitons. The corresponding transmission change spectrum is shown in Figure 13C. Here, PB (transmission increase) is observed on the low-energy side, while PA (transmission decrease) is observed on the high-energy side, indicating blue-shift due to OSE. Note that since this is a transmission change, the sign is opposite to the absorption change shown in Figure 12G. The authors also demonstrated that the blue-shift is maximized when the pump polarization aligns with the direction of the exciton.

Next, we explore probe-polarization-dependent studies. Sim et al. extended their investigation beyond the pump-polarization-dependent exciton OSE in ReS_2_ discussed above, to probe-polarization-dependent effects [237]. As shown by the green lines in Figure 13D, OSE is a coherent phenomenon that occurs only when both pump and probe overlap temporally, enabling ultrafast optical switching. However, even in non-resonant excitation, some carriers can be generated by pump, leading to a slow incoherent dynamics (red lines in Figure 13D), which reduces the overall operational speed. The authors noticed that at specific probe angles, the slow incoherent component becomes nearly zero (third panel in Figure 13D), providing a principle to suppress unwanted slow components by tuning the polarization.

The method of fixing the pump and probe polarizations while rotating the sample has also been employed to study anisotropic exciton dynamics [238], [239]. As an example, Jiang et al. utilized this approach to investigate monolayer ReSe_2_ by keeping the pump and probe polarizations perpendicular and rotating the sample (Figure 13E and F) [239]. Both the pump and probe used 800 nm pulses. Interestingly, an anisotropic dip caused by PB was observed near zero time delay, and its amplitude was maximized when the probe polarization was perpendicular to the *b*-axis. It was attributed to the anisotropic electronic density of states, which leads to a more efficient PB in the direction perpendicular to the *b*-axis.

Finally, we discuss the decay dynamics of excitons in A2DMs. Following exciton formation, its density decreases through various routes such as defect trapping [200], [240], [241], [242], direct radiative recombination [243], and indirect phonon-assisted recombination [244]. In particular, an Auger-type process known as exciton–exciton annihilation (EEA) plays a significant role in the rapid depopulation of excitons in 2D materials. In this process, two excitons collide, with one undergoing nonradiative recombination and the other being excited to a higher state. Surrente et al. investigated the onset density of EEA in monolayer BP through TRPL [245]. They observed a growing EEA-induced fast decay component as the pump fluence increased (Figure 13G), revealing that EEA becomes the dominant recombination mechanism at exciton densities exceeding 6.1 × 10^12^ cm^−2^. Pareek et al. used TA microscopy to explore the unique dimensionality of EEA in bilayer BP [246]. Due to the smaller effective mass of excitons in the AC direction compared to that along the ZZ direction, excitons diffuse more effectively in the AC direction. Thus, EEA in BP shows 1D-like behaviors. Interestingly, it transitions to 2D characteristics as exciton density increases, and then reverts to 1D-like characteristics with decreasing temperature (Figure 13H). These results unveil the distinct dimensionality transition of exciton–exciton interactions in the anisotropic 2D system. EEA has also been observed in ReS_2_. Sim et al. observed that EEA exhibits lower thickness dependence compared to other isotropic 2D semiconductors, attributing this to relatively weak interlayer coupling preventing substantial changes in the electronic structure with thickness [247].

### 4.3 Anisotropic diffusion of carriers and excitons

**Figure 14:**
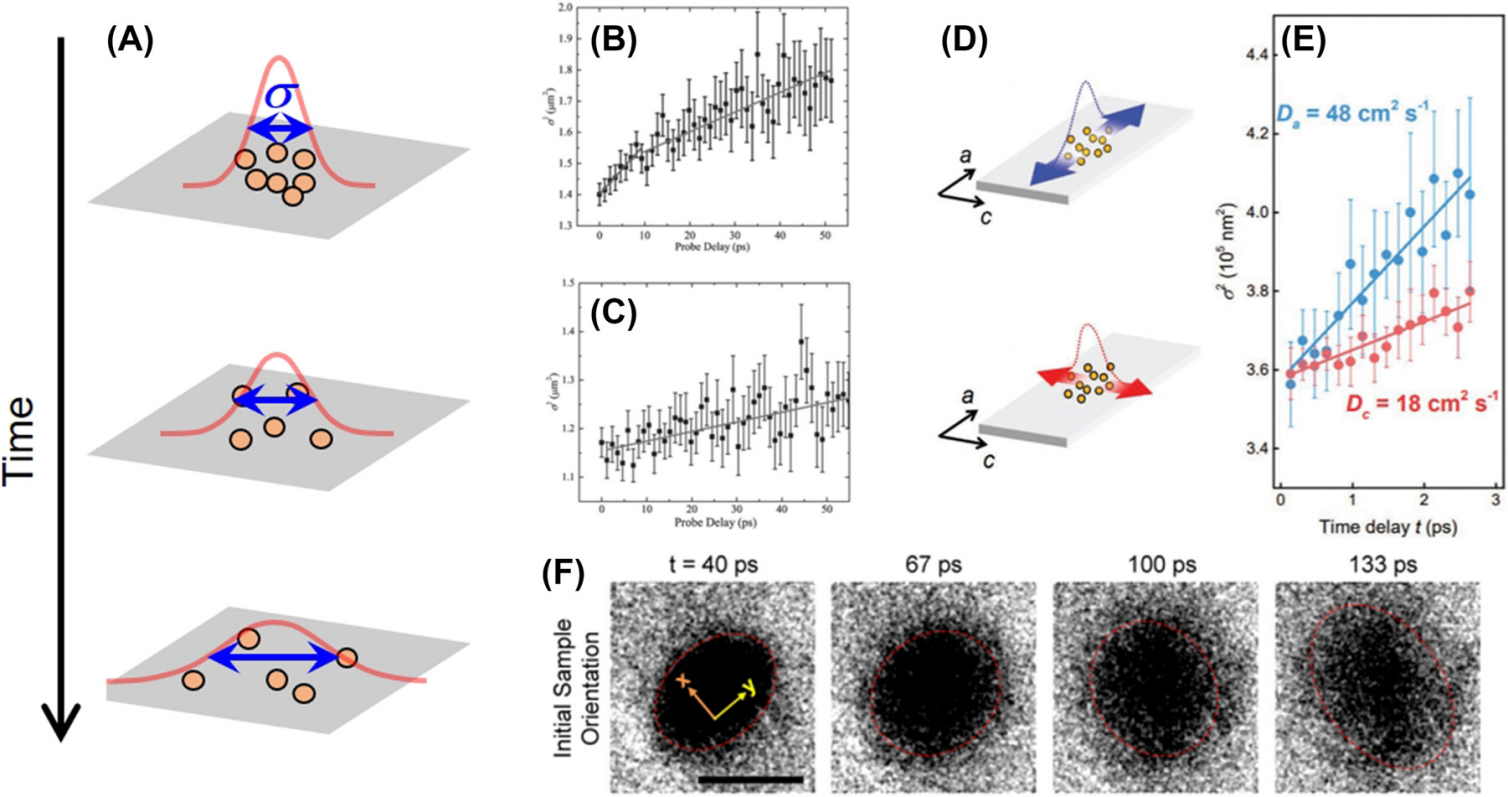
Diffusion dynamics of charge carriers and excitons. (A) Carriers diffusing spatially over time, where *σ* denotes the width of the spatial distribution of carriers/excitons at a specific moment. (B–C) Temporal evolution of squared width (*σ* ^2^) of exciton distribution in monolayer ReS_2_ in the directions parallel (B) and perpendicular (C) to the *b*-axis. Reprinted with permission from [238] © 2015 Wiley-VCH Verlag GmbH & Co. KGaA, Weinheim. (D–E) Diagrams illustrating the diffusion of photo-excited carriers in ZrTe_5_ in two perpendicular directions (D) and the corresponding direction-dependent dynamics of *σ* ^2^ (E) [180] © 2022 Wiley-VCH GmbH. (F) Scanning ultrafast electron microscopy images of hole diffusion dynamics in BP. Reprinted with permission from [266]. Copyright 2017 American Chemical Society.

Charge carriers and excitons generated by optical excitation undergo spatial diffusion over time. Observing and understanding such spatiotemporal dynamics not only provide fundamental insights into the behavior of photo-carriers but also offer essential information for device design, such as diffusion coefficients and lengths related to of electrode and layer spacing [248], [249]. Therefore, various experimental methods have been employed to directly detect the spatio-temporal diffusion of carriers and excitons, including TA-based techniques (both beam-scanning and wide-field imaging types) [250], [251], TRPL-based methods [252], [253], and time-resolved scanning electron microscopy (SEM)-based approaches [254]. Through these experiments, changes in the spatiotemporal distribution of carriers are obtained, as illustrated in Figure 14A. The Gaussian width (*σ*) of the spatial distribution can be extracted at each time point. Since the carrier distribution changes over time, $\sigma \left(t\right)$ is also obtained as a function of time. The spatiotemporal diffusion of carriers in 1D space can be described by [255], [256]

(6) $$\frac{\partial n}{\partial t}=D\frac{\partial^{2}n}{\partial x^{2}}-\frac{n}{\tau },$$

where *D* represents the diffusion coefficient, and *τ* is the carrier lifetime. Assuming a Gaussian distribution for carriers, $n\left(x,t\right)\propto \exp\left\{-x^{2}/\sigma {\left(t\right)}^{2}\right\}$ , equation (6) yields the following relationship:

(7) $$\sigma {\left(t\right)}^{2}={\sigma_{0}}^{2}+4Dt.$$

This indicates that the squared width ( $\sigma {\left(t\right)}^{2}$ ) increases linearly with time (*t*), with a slope of 4*D*. Therefore, by linearly fitting the measured $\sigma {\left(t\right)}^{2}$ versus *t* data, the diffusion coefficient can be determined. However, recent experiments have revealed various forms of $\sigma {\left(t\right)}^{2}$ evolution beyond simple linear increase over time, including sub-linear *t*-dependence [257], super-linear *t*-dependence [258], shrinkage [259], oscillation [174], as well as combination of multiple incremental/decremental components [260], [261], [262]. A more comprehensive discussion on these diverse diffusion behaviors, including results from isotropic 2D materials, can be found in recent relevant review articles [255], [256]. Instead, here we focus on the observed anisotropic diffusion in A2DMs.

In A2DMs, direction-dependent in-plane diffusion of carriers and excitons has been primarily investigated. Cui et al. investigated anisotropic exciton diffusion in monolayer ReS_2_ using TA-type spatiotemporal imaging [238]. They compared the exciton diffusion in the direction parallel to the Re-chain (*b*-axis direction) and in the perpendicular direction. In the *b*-axis direction, the slope of $\sigma {\left(t\right)}^{2}$ changes around 10 ps (Figure 14B). The fast diffusion component before 10 ps was attributed to hot exciton diffusion with a diffusion coefficient of $∼40\;\text{c}{\text{m}}^{2}\;{\text{s}}^{-1}$ , which is more than twice the value of the diffusion coefficient after 10 ps. Similar rapid initial diffusion due to hot carriers and excitons has also been observed in other metallic and semiconducting materials recently [260], [261], [262], [263], [264]. In contrast, exciton diffusion in the direction perpendicular to the *b*-axis did not exhibit such rapid dynamics and showed a relatively low diffusion coefficient of $∼5\;\text{c}{\text{m}}^{2}\;{\text{s}}^{-1}$ (Figure 14C). These results directly reveal the anisotropic nature of exciton diffusion dynamics in ReS_2_. Seo et al. performed spatiotemporal TA measurements on quasi-1D ZrTe_5_ nanoribbons [180]. They compared the diffusion along the long axis (*a*-axis; Zr–Te chain direction) and the perpendicular direction (*c*-axis) of the ribbons (Figure 14D). The resulting time evolution of $\sigma {\left(t\right)}^{2}$ is shown in Figure 14E, where the diffusion coefficient in the *a*-axis direction (blue) is observed to be approximately 2.7 times larger than that in the *c*-axis direction (red). This is attributed to the fact that the effective electron mass in the *a*-axis direction is about 2.5 times smaller than in the *c*-axis direction in ZrTe_5_, resulting in an anisotropy in carrier mobility and diffusivity. Direction-dependent ultrafast diffusion has also been investigated in quasi-1D TiS_3_ nanoribbons; however, a significant anisotropy was not observed [265].

Direction-dependent ultrafast carrier diffusion has also been observed in BP. He et al. conducted space-resolved TA measurements and observed a significantly larger carrier diffusion coefficient in the AC direction ( $∼1300\;\text{c}{\text{m}}^{2}\;{\text{s}}^{-1}$ ) compared to the ZZ direction ( $∼80\;\text{c}{\text{m}}^{2}\;{\text{s}}^{-1}$ ), revealing an approximately 16-fold difference [221]. This substantial anisotropy is much greater than the previously observed mobility anisotropy in BP using electrical measurements and is in excellent agreement with theoretical predictions. This finding underscores advantages of all-optical approaches over conventional electrical methods, which can be influenced by external factors such as device fabrication quality. The anisotropy in carrier diffusion in BP has also been observed by Liao et al. using ultrafast SEM imaging [266]. The spatial carrier distribution in the resulting scanning images reveals an elongated elliptical shape in the AC direction (*x*-direction in Figure 14F) as carriers diffuse. The authors deduced from this observation that the diffusion coefficient of hot holes in the AC direction is 15 times greater than that in the ZZ direction.

### 4.4 Symmetry switching

The optical anisotropy of anisotropic 2D materials arises from the low-symmetry atomic structures they possess. Therefore, manipulating the structural symmetry can lead to the modulation of optical anisotropy. One approach to control symmetry involves applying laser pulses, which provides a principle for modulating or inducing transient anisotropic optical properties on ultrafast timescales. In this section, we review recent studies on phot-induced ultrafast symmetry switching conducted in A2DMs.

**Figure 15:**
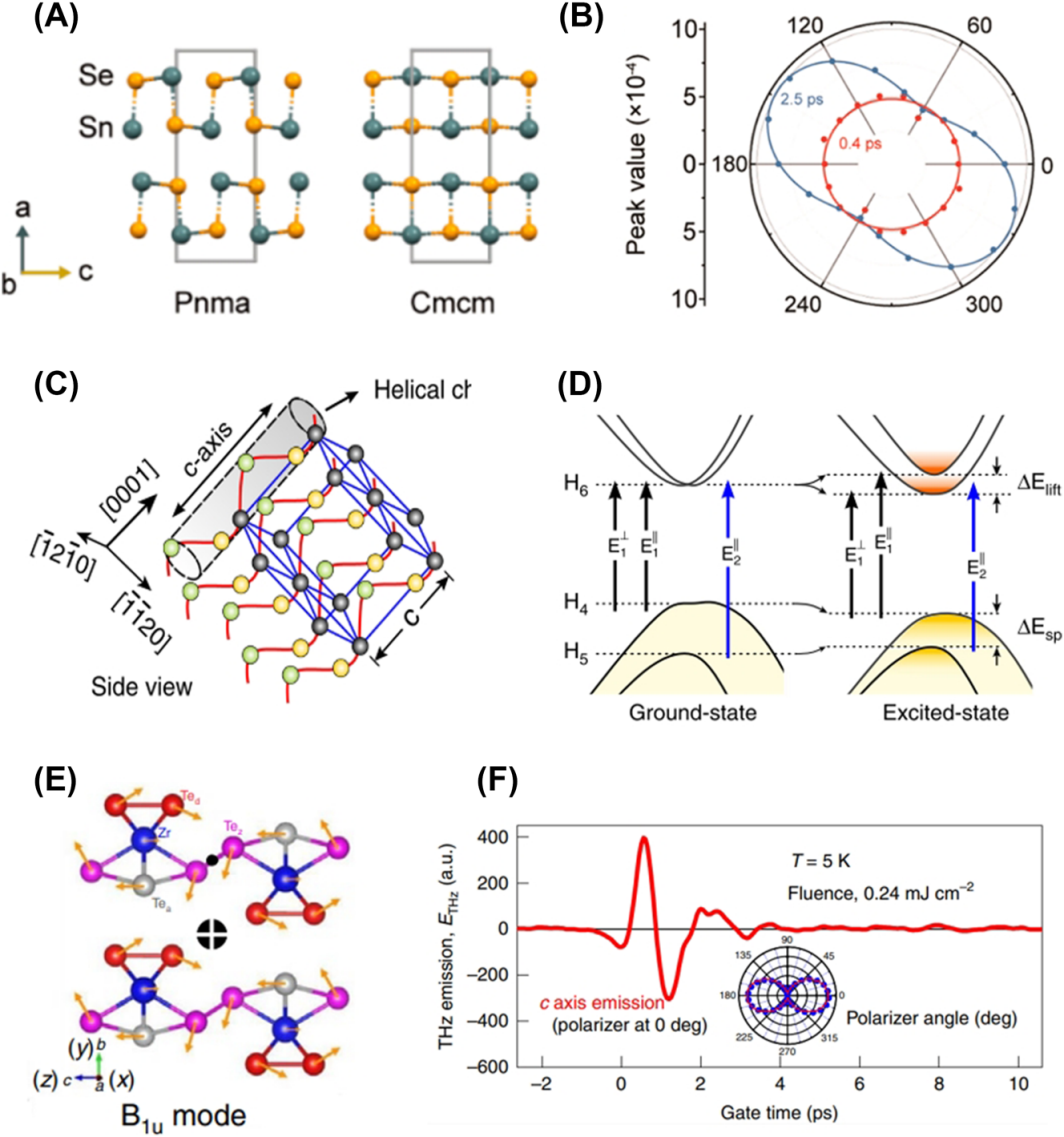
Photo-induced symmetry switching. (A–B) Side-view structures of the Pnma and Cmcm phases in SnSe (A) and polarization-dependent differential reflection patterns in the Pnma (blue dots) and Cmcm (red dots) phases. Reprinted with permission from [267]. Copyright 2022 American Chemical Society. (C–D) Crystal structure of Te (C) and its electronic band structures and interband transitions in the ground state and excited state (D) [270] © (CC-BY 4.0). (E–F) Illustration of the atomic structure and the broken symmetry phonon mode B_1u_ in ZrTe_5_ (E) and its anisotropic THz emission under optical excitation (F). Reprinted figure with permission from [113] © 2021 Springer Nature Limited.

SnSe exhibits a low-symmetry orthorhombic Pnma phase due to Peierls distortion at room temperature, but transitions to a high symmetry Cmcm phase above 800 K (Figure 15A) [267]. Han et al. induced a phase transition in SnSe at room temperature solely through optical pulse excitation [267]. A 1.55 eV excitation induced coherent optical phonons, leading to an anti-Peierls distortion and the consequent formation of the high-symmetry Cmcm phase. As a result, time-dependent anisotropy was observed in polarization-dependent differential reflection traces. Immediately after pump excitation at 0.4 ps, the high-symmetry Cmcm phase emerged, resulting in TA signals exhibiting an isotropic polarization-dependent pattern (red dots in Figure 15B). Conversely, at 2.5 ps, the original low-symmetry Pnma phase was restored, leading to the anisotropic pattern (blue dots in Figure 15B). A similar structure, SnS, also exhibited ultrafast photo-induced symmetry transition through polarization-dependent TA spectroscopy [268]. Through a rigorous analysis of the anisotropy in the fast and slow components of relaxation dynamics, they revealed that these are driven by electron–phonon coupling and the recovery dynamics of the photo-induced symmetry change, respectively. More recently, ultrafast electron diffraction was used to study photo-induced ultrafast structural deformation in GeS [269], which possesses a puckered orthorhombic structure similar to SnSe and SnS. Optical excitation induces converse piezoelectric effects and electron redistribution, leading to directly observed anisotropic structural modulation, contracting lattice vectors in the AC direction and expanding lattice vectors in the ZZ direction.

Jnawali et al. demonstrated ultrafast photo-induced symmetry modulation in monoelemental Te crystals using a broadband IR probe [270]. In Te crystals, helices of Te atoms with 3-fold screw symmetry along the *c*-axis form quasi-1D crystals (Figure 15C). Carriers generated by optical excitation screen the built-in electric field, inducing shear strain through the inverse piezoelectric effect. As a result, the screw symmetry is broken, leading to anisotropic changes in infrared transitions on ultrafast time scales. As illustrated in Figure 15D, in the ground state (i.e., in the absence of pump excitation), an interband transition *E*_1_ has the same energy for probe polarization perpendicular to the *c*-axis ( $E_{1}^{⊥}$ ) and parallel to it ( $E_{1}^{\|}$ ). However, under optical excitation, the broken screw symmetry lifts the degeneracy of the associated conduction band (CB), causing a split between the energies of $E_{1}^{⊥}$ and $E_{1}^{\|}$ . Simultaneously, an anisotropic $E_{2}^{\|}$ transition is also modulated. Consequently, interesting probe polarization-dependent dynamics are observed in MIR differential reflection profiles. These changes gradually diminish as the system returns to the ground state after about 30 ps.

The ultrafast photo-induced symmetry switch has also been investigated in anisotropic topological layered material, ZrTe_5_ [113], [271], [272]. As depicted in Figure 15E, the centro-symmetric ZrTe_5_ crystal possesses inversion centers marked by a black dot and a white cross for Te_z_ and Te_d_ pairs [113]. Using THz generation and optical-pump THz-probe spectroscopy on ZrTe_5_, Luo et al. observed that optical excitation induces coherent phonons of the B_1u_ mode, resulting in a twisted motion of the lattice and consequent breaking of the inversion symmetry [113]. The authors demonstrated that this symmetry breaking along the crystal *c*-axis induces a shift photocurrent along the crystal *c*-axis, leading to highly anisotropic, strong THz emission (Figure 15F). Furthermore, they showed that the photo-induced change in symmetry creates topological Weyl points, generating a robust, dissipationless shift photocurrent along the *c*-axis and circular photogalvanic photocurrent.

### 4.5 Coherent acoustic phonon

In the preceding chapter, we reviewed studies on the modulation of atomic structure by optical excitation, some of which were triggered by coherent optical phonons. Another type of coherent phonon, known as coherent acoustic phonon (CAP), has also been extensively studied using ultrafast spectroscopy [273]. In the process of CAP generation, the optical energy of the pump pulse is converted into mechanical energy within the material due to photo-induced stress, leading to periodic oscillations in the pump-probe signal. The corresponding frequency typically lies in the GHz–THz range.

**Figure 16:**
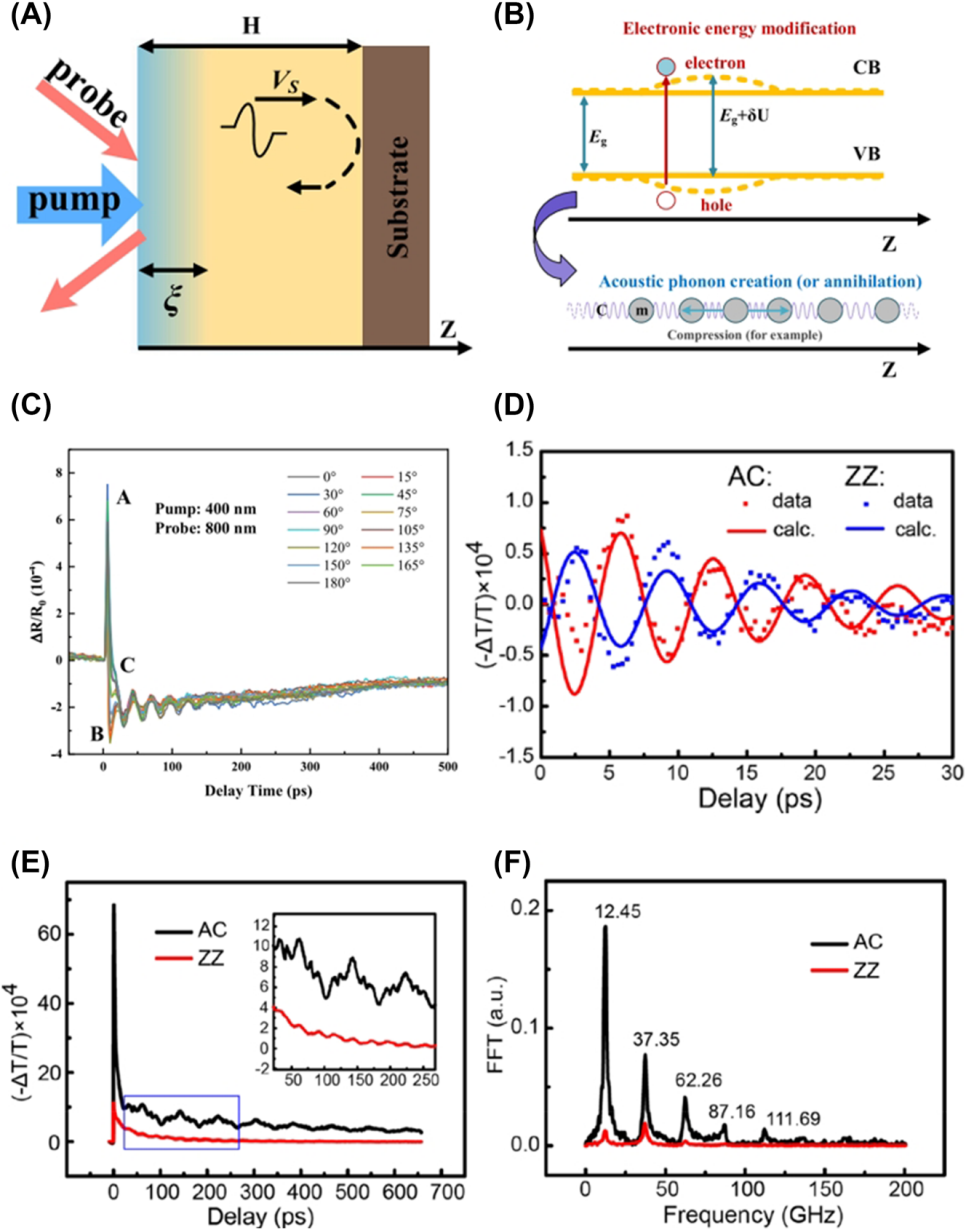
Coherent acoustic phonons (CAPs) in A2DMs. (A–B) Schematic illustration of CAP generation in TA experiments. In (A), *H*, *V*_*s*_, and *ξ* denote sample thickness, sound velocity, and characteristic depth, respectively [274] © (CC BY-NC-ND). (B) Illustration of the deformation potential effect due to pump-generated carriers that induces strain waves. (C) Probe polarization-dependent CAP oscillations in SnS_0.91_Se_0.09_ [274] © (CC BY-NC-ND). (D–F) Anisotropic CAPs in BP. Polarization-dependent CAP oscillations revealing a 180-degree phase difference (D), and polarization-dependent differential transmission traces including high harmonic CAP modes (E) along with their corresponding Fourier spectra (F). Reprinted with permission from [275]. Copyright 2022 American Chemical Society.

The CAP generation process in TA experiments is as follows [273], [274]. As depicted in Figure 16A, a sample material is placed on a substrate, and the pump light impinges normal to it. When the pump pulse reaches the material surface, mechanical strain is induced within a characteristic depth (*ξ*) by various mechanisms, which will be described below. Then, the strain wave generated at the sample surface propagates toward the substrate with the sound velocity (*V*_*S*_). It reflects at the interface between the sample and substrate, traversing back and forth within the sample and prompting localized fluctuations in the dielectric constant. As the probe pulse reaches the sample, a fraction of it gets reflected at the sample surface, while another fraction reflects within the strain wave inside the sample due to the locally altered dielectric constant. These two reflected probe portions may possess different phases, and their phase difference varies periodically over time as the strain wave travels back and forth. Consequently, the interference of these two portions induces periodic oscillations in the probe beam intensity measured at the detector.

The mechanical strain wave is induced by pump via various mechanisms, such as the deformation potential (DP) due to electronic redistribution, the thermoelastic effect due to laser-induced sample heating, electrostriction, inverse piezoelectric effects, and others [273], [274]. Among these effects, the most typically presented in semiconducting systems is the DP effect. When electrons and holes are generated by optical absorption, the local electron distribution is instantaneously modulated, leading to changes in interatomic forces. This results in a transient shift in the lattice equilibrium positions, inducing crystal deformation. As a consequence, strain waves are formed, giving rise to the generation of acoustic phonons (Figure 16B) [274]. These waves then propagate within the sample, leading to periodic oscillations in TA signals. In this DP mechanism, the changes in electron distribution and strain due to lattice deformation are also associated with alterations in the energy levels of specific states, as indicated by the orange dashed lines in Figure 16B.

With this background, let us now discuss the anisotropic CAPs in A2DMs. Anisotropic CAPs, investigated using polarization-dependent TA, have predominantly been observed in materials with puckered orthorhombic structures such as BP [275], SnS [268], and SnS_0.91_Se_0.09_ [274]. Figure 16C displays the probe polarization-dependent differential reflection traces measured in SnS_0.91_Se_0.09_ [274]. Here, oscillating components induced by CAP are superimposed onto background dynamics driven by carriers. The corresponding CAP amplitude is maximized for AC polarization and minimized for ZZ polarization, while the oscillation frequency remains nearly constant across polarizations. The strong polarization dependence of the amplitude and the weak dependence of frequency are behaviors frequently observed in CAPs of other A2DMs [268], [276].

Wu et al. conducted an in-depth investigation of polarization-dependent phase changes and high harmonic modes of CAPs in BP [275]. Figure 16 shows differential transmission traces with probe polarizations in AC (red) and ZZ (blue) directions, where they exhibit a 180-degree phase difference. The pump polarization is fixed in the AC direction. The authors discovered that the differential transmission signal for AC (ZZ) probe polarization is predominantly governed by pump-induced changes in the imaginary part (real part) of the complex refractive index. These responses with different origins for AC and ZZ polarizations undergo opposing changes in response to the strain caused by CAP, resulting in the 180-degree phase difference depicted in Figure 16D. Additionally, the authors observed high harmonic CAP modes. Figure 16E presents CAP traces measured when both pump and probe polarizations are in either AC or ZZ directions. In AC polarization, the trace exhibits a triangular-like oscillation pattern, a result of the overlap of nine higher-order harmonic modes. These modes are also clearly manifested as peak-shaped features in the Fourier-transformed spectrum of the trace (Figure 16F). Conversely, the complex oscillation pattern is not as pronounced for ZZ polarization, mainly due to the contribution of relatively lower-order modes. This study demonstrates that A2DMs exhibit unique anisotropy not only in the amplitude of CAP but also in its phase and higher modes.

## 5 Ultrafast photonics applications

Recent advancements have demonstrated the high potential for various ultrafast photonics applications in 2D materials, including A2DMs, such as all-optical modulators, laser pulse generation, and optical limiting [3], [277]. Their high applicability stems from several key attributes. Firstly, 2D materials exhibit diverse electron structures that enable optical responses spanning a wide spectral range from micrometers to UV wavelengths [3]. Secondly, the enhanced optical nonlinearity arising from the 2D confinement facilitates efficient modulation [277], [278]. Thirdly, strong confinement effects, coupled with reduced dielectric screening, induce robust many-body interactions among fundamental particles, giving rise to diverse exciton complexes that offer a range of mechanisms for ultrafast optical modulation [279], [280]. Finally, the seamless integration of many 2D layers into various structures, such as microcavities, fibers, waveguides, and other 2D materials, eliminates the typical ‘lattice mismatch’ problem encountered with bulk materials [3].

A2DMs retain these typical advantages of 2D materials, while simultaneously offering an additional degree of freedom for polarization-driven ultrafast photonics. In contemporary optical and photonic applications, which encompass optical information, encryption, and communication technologies, the polarization state of light signals serves as a substantial carrier of information [24], [281], [282], [283]. Consequently, there are growing demands for material platforms capable of swiftly processing optical information defined by polarization and efficiently generating polarized ultrafast laser sources. Such demands are particularly pronounced in the steadily increasing utilization of on-chip nanophotonic components [284]. While 1D nanomaterials such as carbon nanotubes exhibit inherent high polarization dependence, they are constrained by practical limitations, such as their smaller cross-sectional area compared to 2D materials and challenges in achieving optically uniform planar structures [285]. In contrast, A2DMs offer a compelling solution with their quasi-1D nature-driven optical responses and high polarization selectivity. These properties, rooted in A2DMs’ unique symmetry without the need for artificial patterning or combinations with other materials, position A2DMs as a promising material platform for polarization-based ultrafast photonics applications.

This section covers ultrafast optical applications based on A2DMs. We now focus our attention on polarization-driven active all-optical modulation and ultrafast pulse generation, areas that have received relatively extensive research attention.

### 5.1 Polarization-driven active all-optical modulation

Photonics technology, encompassing fields like optical computing and optical communication, relies on a combination of various technological elements for the generation, transmission, modulation, detection, and storage of optical signals. Among these, optical modulation stands out as a crucial component of optical processing, involving modulations of information states of light signals [3], [286]. Consequently, various methods, including electro-optic, magneto-optic, and all-optic approaches, have been employed to modulate light signals [3], [287]. Among these methods, the so-called “light-controlled-by-light” or all-optic approach enables ultrafast modulation on picosecond-to-femtosecond timescales, holding the potential to meet the demands for rapid data processing in the era of artificial intelligence and big data [287], [288].

**Figure 17:**
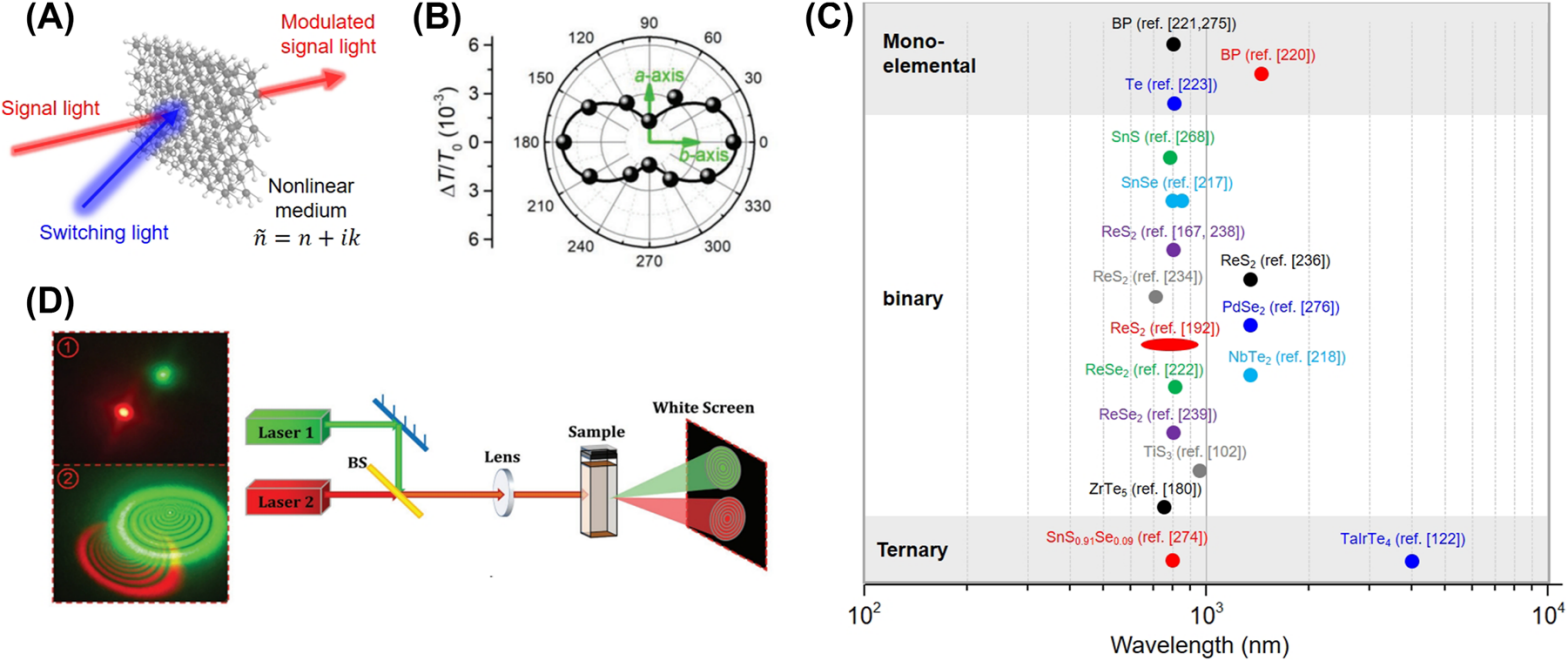
Ultrafast active all-optical modulation using A2DMs. (A) Schematic of ultrafast active all-optical modulation based on a nonlinear medium. (B) Pump polarization-dependent modulation depth obtained from ultrafast all-optical modulation in TiS_3_. Reprinted figure with permission from [102] © 2023 Wiley-VCH GmbH. (C) Operation wavelengths for ultrafast active all-optical modulation with A2DMs in free space (see Table 1). (D) Spatial cross-phase modulation (SXPM) using SnS nano-sheets. Images of the modulated diffraction profiles (left) and experimental configuration (right). Reprinted figure with permission from [293] © 2017 WILEY-VCH Verlag GmbH & Co. KGaA, Weinheim.

The simplified configuration of all-optical modulation is illustrated in Figure 17A [3], [287], [289]. In this setup, the switching light excites a nonlinear medium, modulating its complex refractive index ( $\tilde{n}=n+ik$ ), thereby inducing modulation in the signal light that either passes through or reflects from the nonlinear medium. Changes in the imaginary refractive index (*k*) primarily result in amplitude modulation, while variations in the real refractive index (*n*) are associated with phase modulation [3], [287], [289]. All-optical modulation can further be categorized into active modulation and passive modulation. Active modulation, as depicted in Figure 17A, involves the modulation of signal pulses by different switching lights. In contrast, passive modulation occurs when the signal light self-modulates [287]. A notable application of passive modulation is in ultrafast pulse laser generation based on SA, which we will review later. In this section, our focus is on the state-of-the-art active all-optical modulation using A2DMs.

The simplified structure of active all-optical modulation in Figure 17A closely corresponds to the setup of the TA experiments in Figure 7. Here, the switching light corresponds to the pump pulse in TA, while the signal light corresponds to the probe pulse, and the nonlinear medium represents the sample. Thus, investigations into TA experiments for various materials themselves could be regarded as inquiries into ultrafast all-optical modulation in free space. When A2DMs are employed as the nonlinear medium, their high polarization dependence provides an additional degree of freedom for modulation. Various modulation methods, such as pump polarization, probe polarization, and sample rotation, enable anisotropic modulation. Pump polarization-dependent modulations allow all-optical modulation without altering the intensity of the control light, relying solely on changes in polarization. Probe polarization-dependent modulations enable selective modulation depending on the polarization state of the signal light. Consequently, when the information state of the signal light is defined by its polarization, selective modulation of target information components becomes feasible. This functionality holds the potential for applications in various technologies that utilize optical polarization to convey information, such as polarization division multiplexing, polarization shift keying, and polarization-selective mode shaping [21], [290], [291].

With this perspective in mind, in Table 1, we have summarized the anisotropic TA studies for A2DMs discussed in Section 4 as polarization-driven ultrafast active all-optical modulation. This includes key modulation parameters such as modulation depth, operation wavelength, anisotropy ratio, and recovery time [3]. Modulation depth represents a relative modulation amplitude and can be expressed in various forms. In Table 1, we have listed it as $\frac{\Delta T}{T_{0}}$ or $\frac{\Delta R}{R_{0}}$ , commonly quantities in TA experiments. The operation wavelength corresponds to the wavelength of the modulated light, typically matching the probe wavelength in TA. Note that even in cases where broadband probes were used, we list the specific wavelength if a high response was observed and central analyses were made at that wavelength [102], [180]. The anisotropy ratio is a notation indicating the degree of anisotropy in transmission or reflection modulated with polarization. As an example, Figure 17B shows the peak values of $\frac{\Delta T}{T_{0}}$ measured while rotating the pump polarization in TiS_3_ [102]. Here, it is maximized in the *b*-axis direction and minimized in the perpendicular *a*-axis direction. The anisotropy ratio represents the ratio between these maximum and minimum values, and it is approximately 3.9 in Figure 17B. In A2DMs discussed in this review, similar two-fold behaviors were observed in most cases, with some exceptions [222]. An anisotropy ratio of 1 indicates a complete isotropic response, while a ratio greater than 1 signifies higher anisotropy and greater polarization selectivity. The anisotropy ratio greatly depends on the experimental method (pump polarization rotation, probe polarization rotation, sample rotation), so the used method is also provided in Table 1. Recovery time refers to the time constant of dynamics returning to an unperturbed state after the modulation induced by the pump. It plays a critical role in determining modulation speed and operation bandwidth. These recovery dynamics are influenced by various depopulation mechanisms of charge carriers and exciton, such as defect trapping, Auger recombination, EEA, etc., as discussed earlier.

**Table 1:** Summary of ultrafast active all-optical modulation using A2DMs in free space.

A2DM	Nonlinear medium thickness	Modulation depth (10^−3^)	Operation wavelength (nm)	Rotation	Anisotropy ratio	Time constant	Ref.
BP	80 layers	2.5	1560	Probe-pol.	∼8	∼180 ps	[220]
						∼770 ps	
BP	16 nm	9	810	Sample	∼7	∼100 ps	[221]
BP	15 nm	∼0.4	800	Both pump- and probe-pols.	∼7	1.2 ps	[275]
	9.5 nm	∼0.2			∼10	230 ps	
Te	120 nm	∼0.8	820	Sample	∼2	2.5 ps	[223]
						23.6 ps	
SnS	Bulk	∼35	780	Sample	1.2	∼0.6–1.8 ps	[268]
				Pump-pol.	5.2	∼4–25 ps	
SnSe	175 nm	∼0.25	800	Pump-pol.	4.6	380 ps	[217]
		∼0.33	850		1.6	320 ps	
ReS_2_	Monolayer	∼2	810	Sample	∼11–13	∼10 ps	[238]
						∼40 ps	
ReS_2_	5.6 nm	∼60	810	Pump-pol.	∼5	Sub-ps	[167]
			795		∼3		
ReS_2_	4.5 nm	∼6	730–950	Probe-pol.	∼2	∼1–2 ps	[192]
						∼230 ps	
ReS_2_	37 nm	–	711	Pump-pol.	1.9	0.6 ps	[234]
						2.1 ns	
ReS_2_	3.6–49 nm	–	1040	Pump-pol.	∼1.26	∼5–34 ps	[236]
						81 ps–2.5 ns	
ReSe_2_	Bulk	∼0.8	820	Sample	4-fold pattern	∼1 ps	[222]
						∼60–80 ps	
ReSe_2_	Monolayer	∼0.25	800	Sample	∼6–7	∼33–38 ps	[239]
PdSe_2_	10.6 nm	–	1040	Both pump- and probe-pols.	∼3–4	∼12 ps	[276]
						∼3 ns	
						∼5 ns	
NbTe_2_	15–50 nm	–	1040	Pump-pol.	∼1.1–1.2	∼12–97 ps	[218]
						∼0.15–1.1 ns	
TiS_3_	40 nm	∼2	∼970	Probe-pol.	∼20	0.4 ps	[102]
				Pump-pol.	∼3.9	Sub-ps	
ZrTe_5_	35 nm	∼40	765	Probe-pol.	Infinite	1.8 ps	[180]
TaIrTe_4_	100 nm	∼20	4000	Probe-pol.	∼3	∼1 ps	[122]
						∼3–4 ps	
						∼13–14 ns	
SnS_0.91_Se_0.09_	Bulk	∼0.8	800	Probe-pol.	5.8	>600 ps	[274]

Table 1 displays high anisotropy ratio values from various materials, highlighting the distinctive polarization-based controllability of A2DMs. In particular, ZrTe_5_ exhibited an infinitely large anisotropy ratio at a certain wavelength, as the modulation almost completely disappears when the polarization rotates [180]. Also, many A2DMs in Table 1 demonstrate fast recovery times on sub-picosecond to picosecond scales. However, several limitations are identified. Firstly, the operation wavelength is limited (Figure 17C). In many cases, modulation was only observed at a single wavelength, typically the fundamental output wavelength of the laser source (around 800 nm or 1040 nm), or at specific wavelengths determined by material’s exciton or interband absorption resonances. Secondly, the overall modulation depths are relatively low. It is known that such direct absorptive modulation in all-optical methods is generally lower compared to other techniques like electro-optics and heavily relies on the modulation configuration [3], [287]. Moreover, modulation depth is influenced by various factors such as material thickness, absorption coefficient, pump fluence, and is interconnected with other modulation parameters. For example, increasing material thickness typically extends the interaction distance with light, allowing for higher modulation depths, but this can lead to increased insertion loss or reduced modulation speed [3]. Boosting control light power can enhance modulation depths, but laser-induced damage imposes constraints. Typically, femtosecond laser pulses on 2D materials have a damage threshold ranging from tens to hundreds μJ cm^−2^ [3].

While Table 1 presents cases using short pulses, research employing continuous-wave is also capable of achieving efficient all-optical modulation [139], [217]. The entries in Table 1 correspond to intensity modulation of the signal light, primarily driven by changes in the imaginary part of the complex refractive index (*k*). Research is also actively exploring methods centered on manipulating the real part (*n*) of the material’s refractive index, known as all-optical active phase modulation. Recently, there has been interest in directly detecting changes in *n* and the phase change at ultrafast timescales through frequency domain interferometry-based pump-probe experiments [292]. In addition, spatial cross-phase modulation (SXPM) offers an efficient all-optical active modulation based on phase manipulation. The left images in Figure 17D illustrate how 633 nm light passing through a quartz cuvette containing a dispersion of SnS nanosheets is efficiently modulated by 532 nm control light through SXPM [293]. Furthermore, this spatial modulation principle has been demonstrated for an all-optical information-carrying and conversion system to transmit and convert information.

### 5.2 Ultrafast pulse generation

Pulse lasers find extensive applications ranging from fundamental research to telecommunications, industrial materials, and medical technology [8], [287]. A2DMs, leveraging their nonlinear optical properties, have been intensively employed in both fiber lasers and solid-state bulk lasers for ultrashort pulse generation. The commonly used A2DM-based methods for pulse generation are passive mode locking and passive Q-switching, where A2DMs typically serve as SAs [8]. In passive mode-locked lasers, when pulses initially formed inside the cavity pass through the SA, the leading edge with lower intensity is significantly absorbed compared to the high-intensity peak portion. If the SA’s recovery is fast, even the trailing wing of the pulse can experience substantial absorption. This contribution from the SA helps reduce the pulse duration. Simultaneously, it efficiently absorbs the noise portion of weak intensity. The shortened pulses undergo amplification as they traverse the gain medium, and this process repeats through round trips. Consequently, pulses generated by passive mode locking typically have durations ranging from picoseconds to femtoseconds. The usual repetition rates fall within the MHz to GHz range, determined by the cavity length [8]. On the other hand, passive Q-switching lasers typically operate with tunable repetition rates in the KHz range. In the Q-switching process, the cavity energy continually grows within the gain medium pumped until the SA saturates. As the photon flux inside the cavity increases, the SA approaches saturation. Once the SA reaches saturation, the cavity gain surpasses losses, resulting in the emission of a strong laser pulse [8].

**Figure 18:**
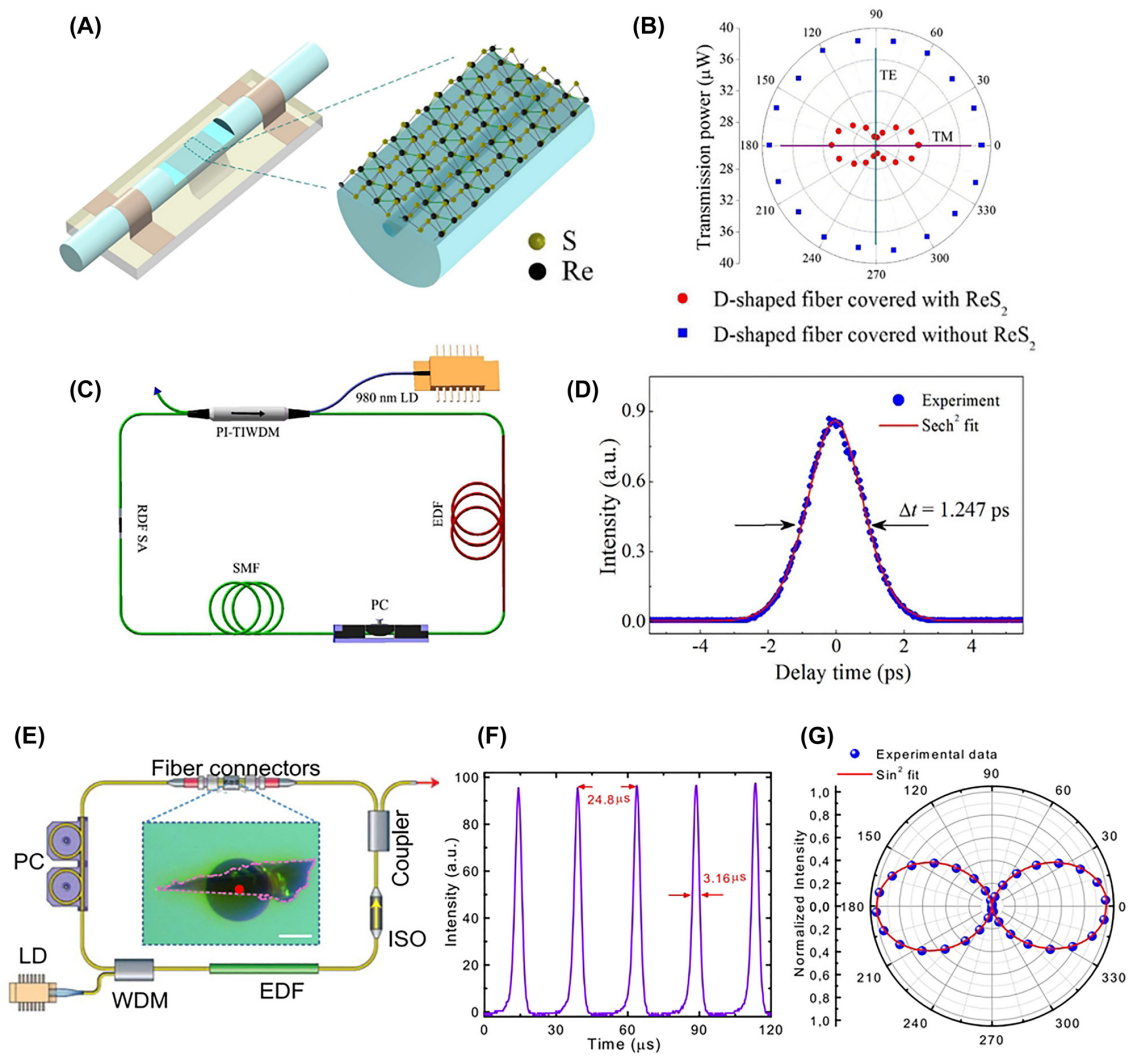
Ultrafast laser pulse generation using A2DMs. (A–D) ReS_2_-based generation. D-shaped fiber SA covered by ReS_2_ (A) and its polarization-dependent transmittance (B). Mode-locked fiber laser configuration (C) and the autocorrelation trace of its output pulse (D) [294] © (CC-BY 4.0). (E–G) BP-based generation. Q-switched fiber laser setup schematic based on BP SA (E), and its output pulses (F) and polarization-dependent intensity (G) [138] © (CC-BY 4.0).

There are various methods to incorporate 2D material SAs into solid-state and fiber lasers. Among these, we briefly review applications that leverage the anisotropic polarization-dependent properties of A2DMs. Cui et al. transferred chemical vapor deposition ReS_2_ onto a D-shape fiber to create an SA, coupling it into the fiber system [294]. The light passing through the SA exhibited high polarization dependence (red dots in Figure 18B), contrasting with the isotropic transmittance without ReS_2_ (blue dots in Figure 18B). Here, transverse magnetic and transverse electric polarizations denote directions parallel and perpendicular to the ReS_2_ plane, respectively. They also demonstrated a mode-locked pulse laser with an erbium-doped fiber (EDF) gain medium using the ReS_2_-covered D-shaped fiber SA. The anisotropy of ReS_2_ resulted in sensitivity to the state of the polarization controller (PC in Figure 18C). With an appropriate PC state, they obtained output pulses with a duration of 1.247 ps and a repetition rate of ∼3.43 MHz (Figure 18D). Liu et al. transferred BP crystals to the end of an optical fiber to fabricate an SA, using it to demonstrate a Q-switched fiber pulse laser operating near the 1550 nm telecommunication band [138]. This produced pulses with durations ranging from ∼9.5 to 3.1 μs (Figure 18F), tunable repetition rates of ∼26–40 kHz, and a maximum output power of over 18 nJ. Remarkably, due to BP’s unique anisotropy, the output displayed fully polarized pulses, as shown in Figure 18G. These authors also utilized BP SA to prove the generation of mode-locked pulses with a duration of ∼786 fs, which also exhibited a high degree of polarization of over 98 % due to BP’s anisotropic characteristics. This result suggests that A2DMs-based SAs hold promise for compact pulse laser generation systems with polarized output, without the need for additional polarization components. Such polarized pulse sources have wide applicability in various fields, including polarized-driven optical switching, coherent Raman scattering, and optical control of lattice vibrations [295]. Furthermore, the high optical anisotropy of A2DMs is expected to be possibly utilized for modulating pulse duration. It is known that the orientation control of phototropic centers (i.e., resonantly absorbing dipoles) in anisotropic SA crystals can be used to modulate pulse duration [296]. Hence, one can anticipate effective control of pulse duration through crystal orientation manipulation in A2DM SAs, given their high optical anisotropy.

Besides ReS_2_ and BP, various A2DMs-based SAs have been employed for short pulse generation in fiber and solid-state lasers. Tables 2 and 3 summarize pulse generation and output performance for passive mode-locking and Q-switching methods, respectively.

**Table 2:** Summary of mode-locked pulse lasers with A2DMs.

A2DM	Center wavelength (nm)	3-dB bandwidth (nm)	Repetition rate (MHz)	Pulse duration (ps)	Average power (mW)	Pulse energy (nJ)	Gain medium	Ref.
BP	1030.6	0.11	46.3	<400	32.5	∼0.702	YDF	[309]
	1033.76	0.48	10	3.27	27	2.7	YDF	[310]
	1053.4	4.65	63.3	0.272	820	∼13.0	Yb,Lu:CALGO	[311]
	1063.3	0.05	0.39	386,300	2.63	∼6.74	YDF	[312]
	1064.1	0.288	140	6.1	460	3.29	Nd:YVO_4_	[313]
	1064.4	5.9	16.77	51	18.9	1.13	YDF	[314]
	1067.1	0.12	0.39	68,400	8.62	∼22.1	YDF	[312]
	1067.1	0.11	0.39	77,200	8.525	∼21.9	YDF	[315]
	1085.5	0.23	13.5	7.54	10	∼0.741	YDF	[316]
	1340.7	0.275	58.14	9.24	350	∼6.02	Nd:GdVO_4_	[317]
	1533	3.7, 6.9	20.821490	–	–	–	EDF	[318]
	1555	4.6	37.8	0.687	1.41	∼0.0373	EDF	[319]
	1555	40	23.9	0.102	1.7	0.071	EDF	[320]
	1556.2	2.2	3.48	1.17	92–145	5.4	EDF	[321]
	1557.2, 1557.7, 1558.2	1	1.65	9.41	21	12.7	EDF	[322]
	1557.8	2.6	6.317	1.2	–	–	EDF	[323]
	1558	6.9	20.822133	–	–	–	EDF	[318]
	1558	7.1	20.82149	0.7	1.5	0.07	EDF	[318]
	1558.14	1.25	15.59	2.18	0.0776	∼0.00498	EDF	[324]
	1558.7	6.2	14.7	0.786	1.6	∼0.109	EDF	[138]
	1558.85	14.2	3.82	0.805	1.412537545	∼0.370	EDF	[325]
	1559	4.5	13.8	0.65	1.7	∼0.123	EDF	[326]
	1559.5	3.8	8.77	0.67	5	∼0.570	EDF	[327]
	1560	8	15.2	0.58	–	–	EDF	[328]
	1560	0.75	1.88	3.6	17.9	9.5	EDF	[329]
	1560.5	10.2	28.2	0.272	0.5	∼0.0177	EDF	[134]
	1560.7	6.4	6.88	0.57	5.1	0.74	EDF	[330]
	1561	0.985	1.025	2.66	1.224	∼1.19	EDF	[331]
	1562	4.5	12.5	0.635	–	–	EDF	[332]
	1562.8	10.7	10.36	0.291	–	–	EDF	[333]
	1564.6	5.7	3.47	0.69	–	–	EDF	[334]
	1566.5	3.39	4.96	0.94	–	–	EDF	[335]
	1566.9	5.6	30.3	–	0.3	∼0.0099	EDF	[336]
	1567.2	5.35	30.3	0.5379	0.33	∼0.0109	EDF	[336]
	1567.5	2.4	15.22	1.08	–	–	EDF	[337]
	1568.19	0.52	1.843	117,600	2.5	∼1.36	EDF	[338]
	1569.24	9.35	60.5	0.28	–	–	EDF	[339]
	1571.45	2.9	5.96	0.946	–	–	EDF	[340]
	1576.1	6.65	34.27	0.4037	1.9	0.055	EDF	[314]
	1601.7, 1602.3, 1602.9, 1603.5, 1604.1	–	1	3.46	9.89	9.89	Zr-EDF	[341]
	1898	3.9	19.2	1.58	2.5	∼0.130	THDF	[342]
	1910	5.8	36.8	0.739	1.5	0.0407	TDF	[343]
	1954.5	3	11.76	1.34	0.5	∼0.0425	THDF	[344]
	2094	4.2	29.1	1.3	11	0.379	HDF	[345]
	2771.1	4.9	27.4	–	6.2	∼0.226	Er:ZBLAN fiber	[346]
	2783	2.8	24	42	613	25.5	Er:ZBLAN fiber	[347]
	2866.7	4.35	13.987	8.6	87.8	6.28	Ho^3+^/Pr^3+^ co-doped fluoride fiber	[348]
	3489	4.7	28.91	–	40	∼1.38	Er:ZBLAN fiber	[349]
Te/BP	1049.1	3.28	42.1	404	292	6.94	Yb:KYW	[350]
Se-doped BP	1579.4	5.8	12	0.686	0.77	∼0.0641	EDF	[351]
Polyimide-BP	1561	2.4	5.268	1.438	5	∼0.949	EDF	[352]
Polyvinyl alcohol-BP	1562	3	5.426	1.236	3	∼0.553	EDF	
b-AsP	1564.52	3.63	8.49	0.828	7.97	0.934	TDF	[353]
Te	980	–	18.8	1600	360	1.037	EDF	[354]
	1060.16	3.44	4.45	465.6	12.7	2.854	YDF	[355]
	1556.57	3.35	15.45	0.879	0.6–5.3	0.343	EDF	[355]
	1558.5	3.27	8.79	1.39	1.9953	0.61	EDF	[356]
	1563.97	38.63	12.17	0.106	106.6	8.76	EDF	[357]
	1567.2	–	22.85	0.621	3.44	0.15	EDF	[358]
	1573.97	18.13	12.17	5.87	23.61	1.94	EDF	[357]
SnS	1530.6	2.6	5.47	1.29	1–1.6	0.182	EDF	[359]
	1560	5.28	8.37	0.656	135	16.13	–	[360]
SnSe	1963	4.3	24.59	1.2	5–47	1.911	TDF	[361]
GeSe	1908.78	2.3	11.36	1.67	0.1–2.7	0.238	THDF	[362]
GeS	1038.8	–	11.4	560	3.4–8	0.701	YDF	[363]
	1556.92	3.8	9.05	0.854	2.75–13.72	1.516	EDF	
GaTe	1030.72	18.1	11.73	752	6–9	0.781	YDF	[364]
	1530.9	18.1	8.79	0.115	1.78–3.84	0.436	EDF	[364]
	1946.2	–	10.71	1.4	0.86	0.08	THDF	[365]
GeP	1550	4.66	14.82	0.722	1.25–28.8	1.943	EDF	[366]
	1896	–	6.07	6.4	58.4	9.621	TDF	[367]
ReS_2_	1060	4.23	50.71	0.323	350	6.903	Yb:CALGO	[368]
	1556	1.85	5.48	1.6	0.4	0.073	EDF	[369]
	1563.3	8.2	1.78	3.8	–	–	EDF	[370]
	1565	26	16.26	0.435	70	0.257	EDF	[371]
	1565	1	1.896	2.549	12	6.329	EDF	[372]
	1970.65	5.05	26.1	0.893	4.13	0.158	TDF	[373]
ReSe_2_	1064	1.3	6514	29	259	0.02	Ti:sapphire	[374]
	1066.5	–	90.37	2290	500–1100	12.17	YVO_4_/Nd:YVO_4_	[375]
	1561.2	3.4	14.97	0.862	0.5	0.334	EDF	[376]
	1927	4.67	17.57	0.84	12.7	0.723	THDF	[377]
	2012.6	–	203.1	580.5	320	1.58	Tm:YAG	[378]
PdS_2_	1033	3.7	24.4	375	15.7	0.64	YDF	[379]
	1565.8	4.48	12.1	0.803	0.55	0.045	EDF	[380]
PdSe_2_	1067.37	–	3.77	767.7	15.6	4.138	YDF	[381]
	1533.61	3.52	7.71	1.19	5.36	0.7	EDF	[382]
	1555.5	–	125.16	1.4	1.2–6	0.048	EDF	[383]
	1556.51	–	37.54	1.23	1.2–6	0.156	EDF	[383]
	1557	–	12.56	1.31	1.2–6	0.478	EDF	[383]
	1560.7	–	16.29	14.92	46.67	2.86	EDF	[382]
	1561.77	–	20.37	0.3237	3.66	0.18	EDF	[381]
GeAs_2_	1560	8.1	8.19	0.371	0.6–3.2	0.391	EDF	[384]
NbS_2_	1064	–	0.1–1.0	15	127	12.7	Yb:KYW	[385]
	1559.36	3.87	18.18	0.709	23.34	1.28	EDF	[386]
	1565.49	–	22.73	0.753	1.45	0.064	EDF	[387]
	1961.44	–	20.23	5.77	5.56	0.275	TDF	
NbSe_2_	1033	0.155	14.7	380	0.5–8.5	0.587	YDF	[388]
	1036	3.4	14.7	174	–	–	YDF	[389]
	1556	2.45	7.7	0.765	3.25–10.75	1.34	EDF	[388]
	1556.3	5.1	–	0.697	–	–	EDF	[390]
	1558.7	4.69	25.31	1.3	6.93	0.27	EDF	[391]
TiS_3_	1555.34	30.49	76.168	0.14772	3.5–12.67	0.1663	EDF	[392]
ZrTe_3_	1068.77	2.02	10.04	323	3.35	0.334	YDF	[393]
	1562.12	2.07	3.377	1.469	2.5	0.74	EDF	[394]
	1563.7	2.31	5.82	1.44	3.46	0.595	EDF	[395]
	1563.8	3.6	11.72	0.751	1.86	0.159	EDF	[393]
	1907.9	3.2	15.24	1.2	65.1	4.27	TDF	[393]
MoO_3_	1559	3.6	10	1.041	10	–	TDF	[396]
Sn–MoO_3_	1560	6.097	10.14	0.467	10.5	1.0353	TDF	[396]
ZrTe_5_	1563	8	4.89	0.892	–	–	EDF	[397]
HfTe_5_	1562	9	3.79	0.878	–	–		
ZrTe_5_	1065.63	–	69	18.4	2.7	0.04	Nd:YVO_4_	[398]
	1343.33	–	71	17.7	1.4	0.02	Nd:YVO_4_	
	2014	–	56	4	767	13.7	Tm:YAG	[399]
Ta_2_NiS_5_	1029	6.8	37.27	1015	37.9	1.017	YDF	[400]
	1036.6	1.1	18.5	270	1.6–9.9	0.535	YDF	[401]
	1557.7	3.5	7.36	0.781	3.3–7.7	0.977	EDF	[401]
	1569	4.89	2.92	1.45	18.6	6.37	EDF	[400]
Ta_2_NiSe_5_	976	–	41.875	0.356	536	12.8	Yb KGW	[402]

EDF, Erbium-doped fiber; TDF, Thulium-doped fiber; YDF, Ytterbium-doped fiber; THDF, Thulium/Holmium-doped fiber; HDF, Holmium-doped fiber; Zr-EDF, Erbium-zirconia-yttria-aluminum co-doped fiber.

**Table 3:** Summary of Q-switched pulse lasers with A2DMs.

A2DM	Center wavelength (nm)	Repetition rate (kHz)	Pulse duration (us)	Max. pulse energy (nJ)	Gain medium	Ref.
BP	635.4	108.8–409.8	0.383–1.56	27.6	Pr^3+^:ZBLAN fiber	[403]
	639	∼135–172	0.189–∼0.51	104	Pr:GdLiF_4_	[404]
	900	8	0.219	6500	Nd:Gd_3_Ga_5_O_12_	[405]
	1028.2	52.54–58.79	1.9–2.4	25.17	YDF	[406]
	1029	63.9	1.73	90	Yb:LuYAG	[407]
	1038.68, 1042.05	52.52–58.73	1.16–2.05	2.09	YDF	[408]
	1040.54–1044.6	40.4–63	2.5–4.7	141.27	YDF	[409]
	1046	∼78–113.6	0.62–1.2	325.7	Yb:CaYAlO_4_	[410]
	1056.6–1083.3	6–44.8	4–5.6	7.1	YDF	[411]
	1060	∼190–312	0.395–∼0.445	70.4	Nd:GdVO_4_	[404]
	1060	220	0.321	600	Nd:Gd_3_Ga_5_O_12_	[405]
	1060.1	∼15.5–24.59	4.21–∼7.25	404.6	YDF	[412]
	1063.8	∼5.5–∼35.5	12.23–∼24.5	∼40–203.38	YDF	[413]
	1063.8, 1064.1	∼5.5–∼39	13.46–∼27	∼20–216.41	YDF	[413]
	1064	20–30.6	0.4955–1.393	1400	Yb^3+^:ScBO_3_	[414]
	1064.1	∼5.5–∼37.5	15.41–∼24.5	∼50–200.75	YDF	[413]
	1064.7	8.9–32.3	4.93–14.18	450.14	YDF	[415]
	1069.4	8.2–32.9	10.8–17.9	328	YDF	[416]
	1300	175	0.363	900	Nd:Gd_3_Ga_5_O_12_	[405]
	1531.2	35.7–70.6	1.65–6.2	25.2	EDF	[417]
	1532	∼15–40	2.3–∼5.5	2150	Er:YAG	[418]
	1532	∼15–36.3	2.55–∼5.1	2400	Er:YAG	[418]
	1532.5	26–40	3.1–9.5	18.6	EDF	[138]
	1550	31.53–82.85	5.52–9.36	51	EDF	[331]
	1550.9	7.46–28.57	5.35–22.34	6.4	EDF	[419]
	1556.93	5.73–31.07	3.59–25.77	142.6	EDF	[420]
	1560	19.25–37.82	1.77–4.23	47.1	EDF	[421]
	1560	16.72–30.71	1.73–4.44	47.6	EDF	[421]
	1560	65–98.5	4.4–2.24	171.7	EDF	[329]
	1561.9	7.86–34.32	2.96–55	194	EDF	[422]
	1562	9.606–44.72	9.84–40.1	80	EDF	[423]
	1562.35	9.61–44.72	9.8–40.1	81.5	EDF	[424]
	1562.87	6.893–15.78	10.32–39.84	94.3	EDF	[340]
	1563.1	15.76–295.98	0.091–0.8907	21.1	EDF	[425]
	1577.9	9.78–61.25	0.742–3.05	40.8	EDF	[426]
	1595	13.33–26.6	7.11–10.67	468.03	EDF	[427]
	1595	13.33–26.6	7.11–10.67	468.03	EDF	[428]
	1644.88	∼18–34	2.8–∼4.8	10,000	Er:YAG	[429]
	1912	69.4–113	0.731–1.42	632.4	THDF	[430]
	1930	17.7	3.1	680	Tm:CaYAlO_4_	[407]
	1941	15.32–27.82	2.92–4.56	500	TDF	[431]
	1948.2	12.5–28.1	5.6–15.1	154.2	Tm^3+^-doped fiber	[432]
	1954	48.3–52.5	0.66–1.15	11,720	TDF	[433]
	1969, 1979	41–81	0.181–0.72	39,500	Tm:YAP	[434]
	1988	∼11–19.25	1.78–∼4.1	7840	Tm:YAP	[435]
	1991	∼11–17.21	2.25–∼4.6	12,030	Tm:YAP	[435]
	2009	∼6.1–∼11.9	2.9–∼9.1	3320	Tm:YAG	[436]
	2056.5	62.4–128.4	2.83–3.04	6700	Tm, Ho:LuVO_4_	[437]
	2100	∼60–122	0.636–∼0.85	221	Tm:Ho:Y_3_Ga_5_O_12_	[404]
	2411	98–176	0.189–0.396	205	Cr:ZnSe	[438]
	2720	12.6	4.47	480	Er:Y_2_O_3_	[407]
	2771.5	∼8–22.2	3.32–∼11.3	820	Er:ZBLAN fiber	[346]
	2779	39–63	1.18–2.10	7700	Er:ZBLAN fiber	[439]
	2793.8	∼26–41.93	0.9548–∼2.325	4250	Er:CaF_2_	[440]
	2797.7	∼29–50.11	0.9405–∼1.5	2470	Er:CaF_2_	[440]
	2840	60–107	0.359–0.72	7100	Er:Lu_2_O_3_	[441]
	2790.1, 2790.9	∼60.5–77.03	0.702–∼1.5	2.34	Er:SrF_2_	[442]
	2970.3	12.43–62.5	2.41–5.8	4930	Ho^3+^-doped fluoride fiber	[348]
	3462	∼55.5–66.3	2.05–∼3.05	1800	Er:ZBLAN fiber	[349]
Te	980	30	12	10.3	EDF	[354]
Te/Bp	1064.4	∼30–126	0.329–∼2	2480	Nd:YAG	[350]
	1956.6	∼25–83	0.25–∼1.8	12,120	Tm:YAP	
	2792	∼25–151	0.163–∼1.5	1920	Er:YSGG	
SnS	–	36.36–65.19	12.5	7563–7670	–	[360]
SnSe	1932	82.25	0.72	8860	Tm:YAP	[443]
	2758	51.28	0.8	9770	Er:CaF_2_	
GeP	1064	160.1	0.628	1140	Nd:GdVO_4_	[444]
	1994.6	51.1	0.4541	3290	Tm:YAP	
	2789.7	70.1	0.5353	1850	Er:SrF_2_	
ReS_2_	400	147	0.4	2120	Nd:GdLaNbO_4_	[445]
	640	520	0.16	100	Pr:YLF	[368]
	976	151	0.444	5300	Yb:LuYLaVO_4_	[446]
	1060	61.2	0.435	5817	Nd:YSAG	[447]
	1064	644	0.139	186	Nd:YAG	[368]
	1064	275–504	0.1216–0 0.29	130	Nd:YAG	[448]
	1064	130–650	0.16–0.65	186	Nd:YAG	[368]
	1064.5	70–165	0.834–2.6	491	Nd:YAG	[449]
	1318	10–308.4	0.111–0.4	330	Nd:YAG	[448]
	1329	69–214	0.403–1.071	420	Nd:YAG	[450]
	1532	43–64	2.1–7.4	38	EDF	[451]
	1557.3	12.6–19	5.496–23	6280	EDF	[452]
	1991	677	0.415	362	Tm:YAP	[368]
	1991	36–68	0.41–0.985	3620	Tm:YAP	[368]
	2790	24–49	0.508–1.625	1210	Er:SrF_2_	[453]
	2796	47–126	0.324–1.1	825	Er:YSGG	[454]
	2950.5	25.48–91.49	0.676–1.233	1130	Ho,Pr:LiLuF_4_	[455]
	–	56.8–66.52	2.4–9.74	18.88	EDF	[456]
ReSe_2_	1047	52–134	1.56–3.32	13.02	YDF	[457]
	1054.2	31.6–68.7	2.87–3.77	81.62	YDF	[458]
	1064.4	84.16	0.682	1490	Nd:YVO_4_	[459]
	1065	17.89–39.86	2.27–5.92	31	YDF	[460]
	1066.5	204–274	1.08–1.53	2500	Nd:YAG	[461]
	1566	6.64–21.04	4.98–16.5	36	EDF	[457]
	1901	54	527.9	15,960	Tm:YLF	[462]
	1937.8	19.5–89.4	0.9258–1.8	17,600	Er:YAP	[463]
	2054.1	106	0.727	9810	Tm:Y_2_O_3_	[462]
	2796	110–244.6	0.2028–0.6375	2200	Er:YAP	[464]
PdS_2_	1567	17.2–26.0	12.6–4.5	15.1	EDF	[380]
PdSe_2_	1064	164	0.34	2470	Nd:GdLaNbO_4_	[465]
NbSe_2_	1560.38	64.14	1.49	48.33	EDF	[466]
TiS_3_	976	102.2–257.6	0.5065	38.9	EDF	[467]
	1556	13.17–48.45	2.34	67.24	EDF	[468]
ZrSe_3_	1064.7	413.47	0.344	1826	Nd:YVO_4_	[469]
	2790	79.15	0.559	8717	Er:ZBLAN fiber	
ZrTe_3_	1064.1	41.8–45.7	5.31–2.55	62.5–67.7	YDF	[393]
ZrTe_5_	2712, 2731	466	0.169	350	Er:YAP	[470]
Ta_2_NiS_5_	1029	104.6–212.3	1.72–1.13	117.2	YDF	[400]
	1065.17	217.4	0.18	1265	Nd:YAG	[401]
	1561	30.02–137.6	5.8–1.72	72.11	EDF	[400]
	1900	50	0.313	22,000	Tm:BYF	[471]
	1986.64	187.932, 151.115	0.787	6800, 9218	Tm:YAP	[472]
Ta_2_NiSe_5_	793	71	0.74	6350	Tm:YAP	[473]
	1000	65	0.355	2700	Nd:YVO_4_	[402]
	2000	61	0.302	7900	Tm:YLF	
	2800	60	0.28	3800	Er:YSGG	

EDF, Erbium-doped fiber; YDF, Ytterbium-doped fiber; THDF, Thulium/Holmium-doped fiber; TDF, Thulium-doped fiber.

## 6 Conclusion and outlook

In this review, we have discussed the ultrafast optical anisotropic characteristics of A2DMs and their applications. Initially, we reviewed the atomic structural features inherent to various A2DMs, as well as their anisotropic traits in linear and nonlinear optical responses. Subsequently, we discussed the diverse ultrafast anisotropic photo-induced phenomena exhibited by time-resolved spectroscopy on A2DMs. Charge carriers and excitons generated in A2DMs through optical excitation have demonstrated various types of polarization-dependent dynamics and anisotropic spatiotemporal diffusion dictated by crystal orientations. Furthermore, we have reviewed studies on transient structural symmetry changes and anisotropic coherent acoustic phonon modes induced by photoexcitation. These polarization-dependent phenomena offer fresh insights and a wealth of physics in the context of anisotropic ultrafast light-matter interactions within 2D systems. Then, we explored applications where the nonlinear optical properties of A2DMs are utilized, such as in polarization-driven active all-optical switches and laser pulse generations, underscoring the significant potential of A2DMs in nanophotonics applications. Nevertheless, for a more profound comprehension of ultrafast light-matter interactions and the expansion of their practical utility, we suggest considering the following issues.

(1) As introduced in Section 2, numerous types of A2DMs have been recently introduced. Their anisotropic linear optical properties, as well as applications such as polarization-sensitive photodetectors, have been extensively demonstrated. However, research on anisotropic ultrafast optical properties is predominantly focused on a few materials. Exploring a wider range of materials would expand our understanding of ultrafast anisotropic light-matter interactions and uncover new applications.
(2) Ultrafast all-optical modulation using A2DMs offers potential for high-speed photonics technologies driven by light polarization. However, there are still many challenges to address. For instance, the nano-thin thickness of A2DMs is suitable for nanophotonic component integration, but it often results in low modulation depth due to the short interaction length with incident light. Increasing the thickness, on the other hand, frequently leads to a trade-off relationship where insertion loss and recovery time increase [3]. Additionally, the operational wavelength range remains highly limited.
(3) This review primarily focused on individual A2DMs. However, it is worth noting that diverse combinations of anisotropic/isotropic and anisotropic/anisotropic van der Waals heterostructures can offer high functionalities [14]. Furthermore, the continuous synthesis of new types of anisotropic 2D materials is expected. Continuous research on the ultrafast optical properties and photonics applications of these novel materials is essential.
(4) 2D moiré superlattices, formed by stacking atomically thin 2D layers with slight rotational twists or lattice mismatches, have attracted considerable attention due to their fascinating photonic, optoelectronic, superconducting, and ferromagnetic properties [297], [298], [299]. Recent advancements in ultrafast laser spectroscopy have allowed for the direct observation of interlayer exciton formation and diffusion within these structures [300], [301]. While most research on 2D moiré superlattices has focused on isotropic materials like WS_2_ or WSe_2_, noteworthy phenomena have emerged from moiré structures based on A2DMs [302], [303], [304]. Zhao et al. unveiled novel optical transitions in twisted monolayer/bilayer phosphorene heterostructures [303], while Wang et al. demonstrated the tunability of chirality in twisted homostructures of various A2DMs [304]. Additionally, intriguing theoretical predictions have reported, including 1D flat bands in twisted GeSe bilayers [305], anisotropic moiré excitons in twisted SnS [306], and strongly bound 1D excitons and superior thermoelectric properties of twisted phosphorene bilayers [307], [308]. However, as of our knowledge, ultrafast optical studies specific to moiré superlattices based on A2DMs have yet to be reported. We anticipate that future research in this area will unveil new insights into ultrafast anisotropic phenomena, further enriching our understanding of these remarkable 2D materials and their potential applications.
